# An investigation of gene dosage reveals that increased sensitivity to D-cycloserine divergently impacts the transience of heteroresistance in *Escherichia coli*

**DOI:** 10.1128/mbio.01490-25

**Published:** 2025-08-22

**Authors:** Katherine J. Sniezek, Mark P. Brynildsen

**Affiliations:** 1 Department of Chemical and Biological Engineering, Princeton University 6740, Princeton, New Jersey, USA; 2 Omenn-Darling Bioengineering Institute, Princeton University, Princeton, New Jersey, USA.; University of Wisconsin-Madison, Madison, Wisconsin, USA; Emory University Vaccine Center, Atlanta, Georgia, USA

**Keywords:** highest non-inhibitory concentration, heteroresistance stability index, UTI89, UPEC, *cycA*, *ddlB*

## Abstract

Heteroresistance describes the phenomenon where seemingly isogenic bacterial populations contain subpopulations with elevated resistance compared to the susceptible majority that are often missed in routine susceptibility testing. The enhanced resistance of those subpopulations can either be maintained (stable) or quickly lost (unstable/transient) after treatment, where transient cases return susceptibility results identical to those of original cultures. Recent work has implicated increased gene dosage of resistance determinants as a major cause of unstable heteroresistance in clinical isolates. Inspired by that work, we sought to systematically evaluate how gene dosage of an antibiotic’s target network components impacted heteroresistance and its stability. To accomplish that, we used *Escherichia coli* MG1655 as a model organism, and D-cycloserine (DCS), a cell-wall synthesis inhibitor that enters through a transporter (CycA) and inhibits multiple enzymes (DdlA, DdlB, DadX, and Alr), as a model antibiotic. To measure heteroresistance, we used population analysis profiling, and to quantify stability, we used a population-level model to define a heteroresistance stability index (${HSI}_{j}{|}_{t}$), which quantifies the proportion of heteroresistant subpopulation *j* that maintained elevated resistance over *t* generations. We found that increased sensitivity to DCS through gene dosage variation of *cycA* and *ddlB* divergently impacted heteroresistance stability, with *ddlB* enhancing stability and *cycA* fostering transience. These findings translated to uropathogenic *E. coli* (UTI89) and suggested that increasing the number of antibiotic targets and/or points of antibiotic entry could decrease the propensity of heteroresistance to yield stable resistance. This knowledge could impact the development of new antibiotics and improve understanding of antibiotic treatment failure.

**IMPORTANCE:**

Heteroresistance is a concern because heteroresistant strains escape clinical detection and facilitate treatment failure. Heteroresistant cells can produce stably resistant or transiently resistant populations, and enhanced understanding of genetic factors that influence the level of heteroresistance and its stability has the potential to improve treatment strategies. Here, we introduce the heteroresistance stability index, which is a quantitative metric of heteroresistance stability, and use it to analyze heteroresistance of *Escherichia coli* to D-cycloserine. We investigated how gene dosage of antibiotic target network components (transporter, enzymatic targets) influences heteroresistance and its stability and found diverging outcomes on stability for comparable declines in heteroresistance. Specifically, these results suggest that designing antibiotics to enter through multiple transporters or target multiple enzymes would reduce the emergence of stable resistance.

## INTRODUCTION

Antimicrobial resistance (AMR), or the ability of microbes such as bacteria to grow at higher antibiotic concentrations than susceptible standards (as measured by MICs [1]), is a major global health crisis that threatens the efficacy of even last-resort antibiotics (2). Not only did AMR cause an estimated 1.27 million deaths in 2019 (3), but it is also projected that 10 million deaths per year will be attributable to AMR by 2050 if unmitigated (4). To address this crisis, it is critical to understand how antibiotic treatments fail so that we can identify better treatment regimens, slow the emergence of resistance, and improve treatment outcomes (5–9).

One underappreciated and understudied contributor to antibiotic treatment failure and resistance development is heteroresistance (10–12). Heteroresistance is a phenomenon where a population of seemingly isogenic bacteria contains subpopulations with higher MICs relative to the sensitive majority (11, 12). Since its earliest reports, such as that from Alexander and Leidy who were studying streptomycin resistance of *Haemophilus influenzae* in 1947 (13), heteroresistance has been observed to a variety of antibiotics from different classes (e.g., β-lactams [14], carbapenems [15–18], aminoglycosides [18], polymyxins [19–26], fosfomycin [FOS] [27, 28], fluoroquinolones [FQs] [29–31], and glycopeptides [32, 33]) in cultures of Gram-positive (32, 33), Gram-negative (14–28, 30, 34), and mycobacterial (29, 31) isolates. Importantly, resistant subpopulations of heteroresistant infections can escape detection by standard clinical tests (25, 35), and it has been proposed that heteroresistance provides an evolutionary path toward the development of homogeneous resistance (14). For instance, one historical analysis of β-lactam susceptibility among *Escherichia*, *Enterobacter*, and *Klebsiella* clinical isolates revealed that, after a given β-lactam was clinically introduced, a rise in frequency of heteroresistant isolates preceded that of resistant isolates (14), suggesting that heteroresistant isolates could facilitate the emergence of wholly resistant strains (10).

One important feature of heteroresistance is the stability of subpopulation resistance after treatments end (11, 12). It has been observed that the progeny of heteroresistant subpopulations can either inherit and maintain increased MICs in the absence of antibiotic pressure (referred to as stable heteroresistance) or lose their elevated resistance once antibiotic treatment concludes (referred to as unstable/transient heteroresistance) (11, 12). While both stable and transient heteroresistance can threaten treatment success, the latter presents clinical conundrums because the measured MIC of a transiently heteroresistant isolate can be identical before and after treatment (24, 25). For example, Band and colleagues observed that colistin-heteroresistant *Enterobacter cloacae* infections mediated treatment failure in a mouse model despite the isolate being classified as susceptible to colistin by *E*-test both before and after treatment (24). Notably, transience within heteroresistant subpopulations has been difficult to analyze because most subpopulations only partially revert to the drug susceptibilities of their originating cultures (18, 36). Such observations argue that a binary classification system of stable or transient is insufficient to capture the complexities of this phenomenon.

Recognizing the growing incidence and impact of heteroresistance in the clinic, researchers have begun identifying the underlying mechanisms responsible for this phenomenon. Nicoloff and colleagues have reported that increased dosage of AMR genes via chromosomal or plasmid-mediated amplification causes the transient heteroresistance of various Gram-negative clinical isolates to a variety of antibiotic classes (18, 37). Other studies have identified the two-component regulatory system PhoPQ as important for the colistin heteroresistance exhibited by *Klebsiella pneumoniae* (26) and *E. cloacae* (20, 24). However, because heteroresistance has largely been studied in clinical isolates, which are more challenging to genetically interrogate, in-depth investigations into genetic factors that modulate this phenomenon have remained limited. Arguably, uncovering the factors that influence heteroresistance and its stability could provide knowledge to improve antibiotic treatment outcomes.

Here, we sought to analyze genetic factors that influence heteroresistance and its stability. To accomplish that, we needed to identify a genetically tractable strain that exhibits heteroresistance and develop a quantitative framework for rigorous assessment of transience in heteroresistant subpopulations. To begin, we screened *Escherichia coli* K-12 MG1655 for heteroresistance to a variety of mechanistically distinct antibiotics using population analysis profiling (PAP) and identified several to which MG1655 was heteroresistant. Of those antibiotics, we found that MG1655 exhibited the highest degree of heteroresistance toward D-cycloserine (DCS), a cell wall synthesis inhibitor that targets multiple conditionally essential intracellular enzymes (38–41), enters the cytoplasm via a transporter (42), and is indicated as a second-line treatment for tuberculosis and acute urinary tract infections (UTIs) (43). We then developed a metric we call the heteroresistance stability index (${HSI}_{j}{|}_{t}$) based on a population-level model to analyze the stability of heteroresistant subpopulations using PAP data and applied it to examine how gene dosage of DCS network components impacted heteroresistance and its stability. Importantly, we focused on gene dosage due to recent reports implicating it as a mechanism of transient heteroresistance (18, 37, 44, 45). We found that increasing DCS sensitivity through distinct genetic means (deletion of DCS target genes, increase of DCS transporter copy number) divergently impacted the stability of heteroresistant subpopulations, with stable heteroresistance occurring at lower concentrations in strains with fewer DCS targets and transient heteroresistance expanding to higher concentrations in strains with increased transporter copy numbers. This diverging impact of increased sensitivity on heteroresistance stability for DCS was also observed in uropathogenic *E. coli* (UPEC) isolate UTI89. These results show how MG1655 can be used to elucidate genetic factors influencing heteroresistance that are shared with pathogenic isolates. Furthermore, the data and analyses presented suggest that antibiotics with more targets and/or nodes for entry are less prone to contain subpopulations that are stably resistant.

## RESULTS

### *E. coli* MG1655 exhibits heteroresistance to antibiotics with different mechanisms of action

To initiate our investigation into heteroresistance stability and the genetic factors that influence this phenomenon, we assayed *E. coli* MG1655 for heteroresistance to a panel of bactericidal antibiotics with different mechanisms of action using PAP, the gold standard for measuring heteroresistance (11, 12). Briefly, PAP is a colony-counting assay that quantifies the proportion of a bacterial population that can grow on media agar plates supplemented with twofold increasing concentrations of antibiotic relative to an antibiotic-free plate (for additional details, see Materials and Methods). MG1655 was chosen due to its vast knowledge base (46) and genetic tractability (47–49), which would facilitate mechanistic investigations of heteroresistance if parallel phenomena were observed between pathogenic *E. coli* and this model organism, as well as its recent use in investigations of heteroresistance (50). The antibiotic panel included FOS, which disrupts cell wall synthesis by inhibiting MurA, the enzyme responsible for the first dedicated step of peptidoglycan synthesis (51); tobramycin (TOB), a 30S ribosomal subunit inhibitor which causes protein mistranslation (52); ciprofloxacin (CIP), a FQ which primarily targets DNA gyrase in *E. coli* and induces DNA damage (53); and DCS, which also disrupts cell wall synthesis, but through different targets that include D-alanine ligases (DdlA and DdlB) and D-alanine racemases (DadX and Alr) (38–41).

**Fig 1:**
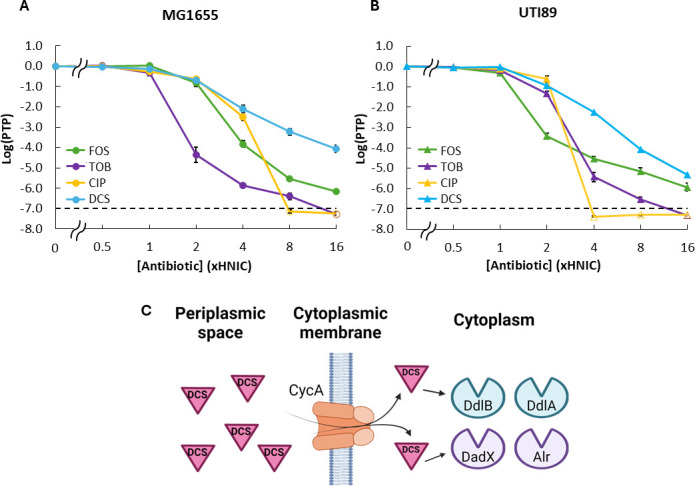
MG1655 and UTI89 exhibit high levels of heteroresistance to DCS. (**A**) MG1655 and (**B**) UTI89 PAP data for various antibiotics. Overnight cultures grown in drug-free media were spotted on drug-free agar plates and agar plates containing twofold increasing concentrations of antibiotic, CFUs were enumerated, and the proportion of the total population (PTP) growing at each antibiotic concentration was calculated. For drugs with no known transporters (CIP and TOB), cation-adjusted Mueller-Hinton agar (CAMHA) media was used. For drugs with known transporters (FOS and DCS), an appropriate medium was selected to ensure expression of the transporter (see Materials and Methods). HNIC indicates the highest drug concentration evaluated during PAP at which there was no statistically significant difference in the log-transformed PTP growing at that drug concentration ($log\left[{PTP}_{1\times HNIC}\right]$) and that on drug-free plates ($log\left[{PTP}_{0\times HNIC}\right]$) by one-way analysis of variance (ANOVA) with Tukey *post hoc* analysis (*P* > 0.05), and the majority of the culture grew (${PTP}_{1\times HNIC}$> 0.5). For MG1655, HNIC_FOS_ = 0.2 µg/mL, HNIC_TOB_ = 0.256 µg/mL, HNIC_CIP_ = 0.002 µg/mL, and HNIC_DCS_ = 0.128 µg/mL. For UTI89, HNIC_FOS_ = 0.4 µg/mL, HNIC_TOB_ = 0.256 µg/mL, HNIC_CIP_ = 0.004 µg/mL, and HNIC_DCS_ = 0.512 µg/mL. Data represent averages of at least three biological replicates, and error bars represent SEM. Unfilled data markers indicate concentrations where subpopulation growth was below the limit of detection for the majority of replicates. The dashed line indicates the threshold for heteroresistance. (**C**) Schematic representation of DCS antibiotic network that includes cytoplasmic membrane transporter CycA and four target enzymes (isozymes DdlA and DdlB, and isozymes Alr and DadX). Created in BioRender (K. Sniezek, 2025, https://BioRender.com/vwa16s1).

A strain is considered heteroresistant if it contains subpopulations that grow at a frequency ≥10^−7^ at antibiotic concentrations at least eightfold higher than the highest non-inhibitory concentration (HNIC; i.e., the highest antibiotic concentration at which the majority of the population can grow, see Materials and Methods) (11, 12). If a strain does not exhibit detectable growth at or above 8× HNIC but does exhibit growth at 4× HNIC, it is considered intermediately heteroresistant (11, 12). Any strain that does not meet these criteria is considered to behave homogeneously for that antibiotic (11). From PAP data, we observed that MG1655 exhibited heteroresistance to FOS, TOB, and DCS and intermediate heteroresistance to CIP (Fig. 1A). We observed similar results for UPEC UTI89, which also exhibited heteroresistance to FOS, TOB, and DCS (Fig. 1B). Notably, these results suggest that MG1655 could serve as a useful model organism to study heteroresistance of UPEC. Of these antibiotics, we chose to further investigate heteroresistance to DCS here for two reasons: (i) MG1655 and UTI89 exhibit the greatest degree of heteroresistance to DCS among the antibiotics assayed (Fig. 1A and B), and (ii) the DCS target network contains a variety of features that can be probed for their impact on heteroresistance stability, which include a transporter (CycA) and four conditionally essential target enzymes (DdlA, DdlB, DadX, and Alr; Fig. 1C). Thus, DCS can serve as a model antibiotic where insights into its heteroresistance may apply to other agents that enter through a transporter (e.g., FOS [42] and cefiderocol [54–56]) and/or have multiple primary targets (e.g., penicillins [57] and FQs [53]).

### DCS heteroresistance exhibits a window of transience with respect to concentration

**Fig 2:**
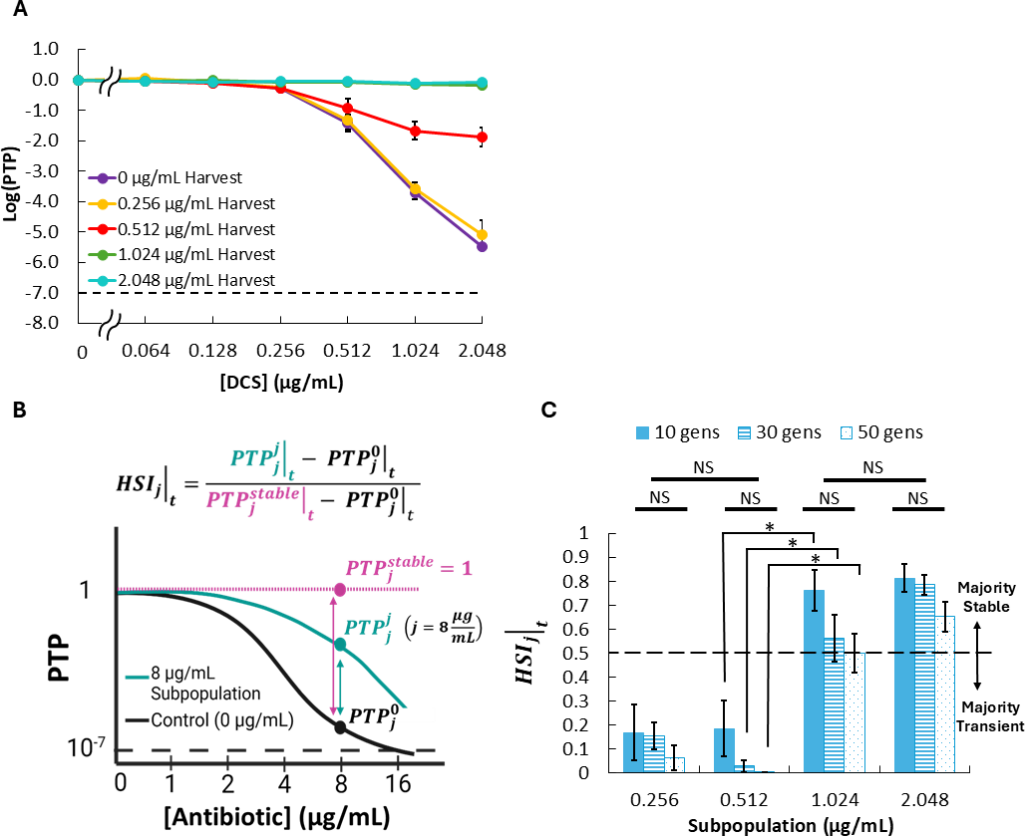
Measuring subpopulation stability of DCS heteroresistant subpopulations of MG1655. (**A**) PAP of harvested DCS-resistant subpopulations after propagating for 10 generations without antibiotics. The dashed line indicates the threshold for heteroresistance. (**B**) Schematic representation of ${HSI}_{j}{|}_{t}$, which quantifies the proportion of a subpopulation harvested from antibiotic concentration *j* that maintains its elevated resistance to the same drug concentration after *t* generations of propagation without antibiotic. Created in part in BioRender (K. Sniezek, 2025, https://BioRender.com/dhbm7yt). (**C**) ${HSI}_{j}{|}_{t}$ for each DCS minority subpopulation after *t* = 10, 30, or 50 generations of propagation. Data represent averages of at least three biological replicates, and error bars represent SEM. Dashed line delineates the point of transition between subpopulation stability states (majority transient for ${HSI}_{j}{|}_{t}<0.5,$ vs majority stable for ${HSI}_{j}{|}_{t}>0.5$). Star represents statistical significance by one-way ANOVA and Tukey post hoc analysis (*P* < 0.05). NS = no significance.

We next probed the stability of each minority subpopulation from DCS PAP assays performed on MG1655. We were interested in quantifying the degree to which each subpopulation collectively maintained its elevated resistance after regrowth without antibiotics. To accomplish that, we harvested minority subpopulations at each drug concentration where growth was detected by PAP ([DCS] = 0.256 µg/mL up to [DCS] = 2.048 µg/mL, in addition to [DCS] = 0 µg/mL as an untreated control), grew them for ~10 generations in LB media, and then re-assayed the harvested subpopulations with PAP. The results for the 0.256 µg/mL subpopulations aligned with that of control cultures, suggesting largely transient resistance, whereas the 1.024 and 2.048 µg/mL subpopulations displayed uniformly resistant profiles, suggesting predominantly stable resistance (Fig. 2A). However, PAP data for the 0.512 µg/mL subpopulations were between the control curve (complete transience) and uniform resistance, suggesting that only a portion of the harvested subpopulations maintained elevated resistance after antibiotic-free propagation. These results underscored the need for a metric to quantify the degree of stability or transience exhibited by heteroresistant subpopulations because that characteristic was not binary (transient or stable) but a mix of states.

To more quantitatively interpret PAP subpopulation stability data, we derived the ${HSI}_{j}{|}_{t}$ metric, which is calculated by:


(1) $${{HSI}_{j}|}_{t}=\frac{{{PTP}_{j}^{j}|}_{t}-{{PTP}_{j}^{0}|}_{t}}{{{PTP}_{j}^{stable}|}_{t}-{{PTP}_{j}^{0}|}_{t}},$$

where ${{PTP}_{j}^{j}|}_{t}$ is the proportion of a subpopulation initially harvested from antibiotic concentration *j* (superscript *j*) that grows at the same concentration (subscript *j*) after *t* generations of propagation in antibiotic-free media; ${{PTP}_{j}^{0}|}_{t}$ is the proportion of the untreated control culture (harvested from antibiotic-free/0× HNIC plates) that grows at concentration *j* after the same time of propagation in antibiotic-free media; and ${{PTP}_{j}^{stable}|}_{t}$ is the theoretical proportion of the subpopulation initially harvested from antibiotic concentration *j* that would grow at concentration *j* if the entire subpopulation maintained its resistance after propagation in antibiotic-free media (i.e., was completely stable), which is assumed to be 1 (see Materials and Methods for derivation). This metric calculates the deviation in a minority subpopulation’s resistance from that of the untreated control relative to the maximum possible deviation from the control at the same concentration from which the subpopulation was initially harvested. In essence, ${HSI}_{j}{|}_{t}$ represents the proportion of subpopulation *j* that maintains its resistance to that same concentration after *t* generations of propagation in antibiotic-free media. Thus, an${HSI}_{j}{|}_{t}$ value of 1 indicates complete subpopulation stability, whereas an ${HSI}_{j}{|}_{t}$ value of 0 indicates no deviation from control, and thus, the resistance of the entire subpopulation would be transient (Fig. 2B). We note that, due to its functional form, the error associated with the ${HSI}_{j}{|}_{t}$ calculation increases as ${{PTP}_{j}^{0}|}_{t}$ approaches a value of 1. Consequently, to ensure robust application of this metric, we confine this framework to evaluate the stability of subpopulations harvested from concentrations on which the control culture produces a minority subpopulation (${{PTP}_{j}^{0}|}_{t}<0.5$) for the majority of replicates.

Applying this calculation to the DCS subpopulation stability data, we found that the resistance of the 0.512 µg/mL subpopulation was predominantly transient, and there is a statistically significant difference in the stability between subpopulations harvested at DCS concentrations < 0.512 µg/mL and those harvested at >1.024 µg/mL (Fig. 2C). The stability profile of each subpopulation was also largely maintained through 50 generations without antibiotic pressure, as there was no significant difference in ${HSI}_{j}{|}_{t}$ measurements for each subpopulation after 10, 30, and 50 generations of propagation (Fig. 2C; Fig. S1). Thus, we see that there is a concentration window that includes 0.256 µg/mL and 0.512 µg/mL DCS where minority subpopulations were transiently resistant, which yields predominantly stable subpopulations at concentrations of 1.024 µg/mL and above.

### Gene dosage of the DCS transporter CycA impacts heteroresistance and subpopulation stability

Given the genetic tractability of MG1655 and knowledge of the DCS target network, we sought to assess whether any of its components impacted heteroresistance to DCS and/or the stability of the heteroresistant subpopulations. We began our network investigation with CycA, the D-serine/alanine/glycine:H^+^ symporter through which DCS enters the cytoplasm (Fig. 1C) (42). Inspired by reports that gene amplifications play a role in transient heteroresistance of Gram-negative isolates (18, 37, 44, 45), we interrogated how gene dosage of this transporter impacted DCS heteroresistance. To do this, we constructed a genetic deletion knockout in MG1655 (Δ*cycA*), as well as an MG1655 strain harboring an additional *cycA* copy inserted at the Tn7 attachment site (*attTn7*) of the chromosome. We expected that decreasing *cycA* copy number (0 copies) would increase resistance to DCS (58), whereas increasing *cycA* copy number (two copies) would decrease resistance to DCS.

**Fig 3:**
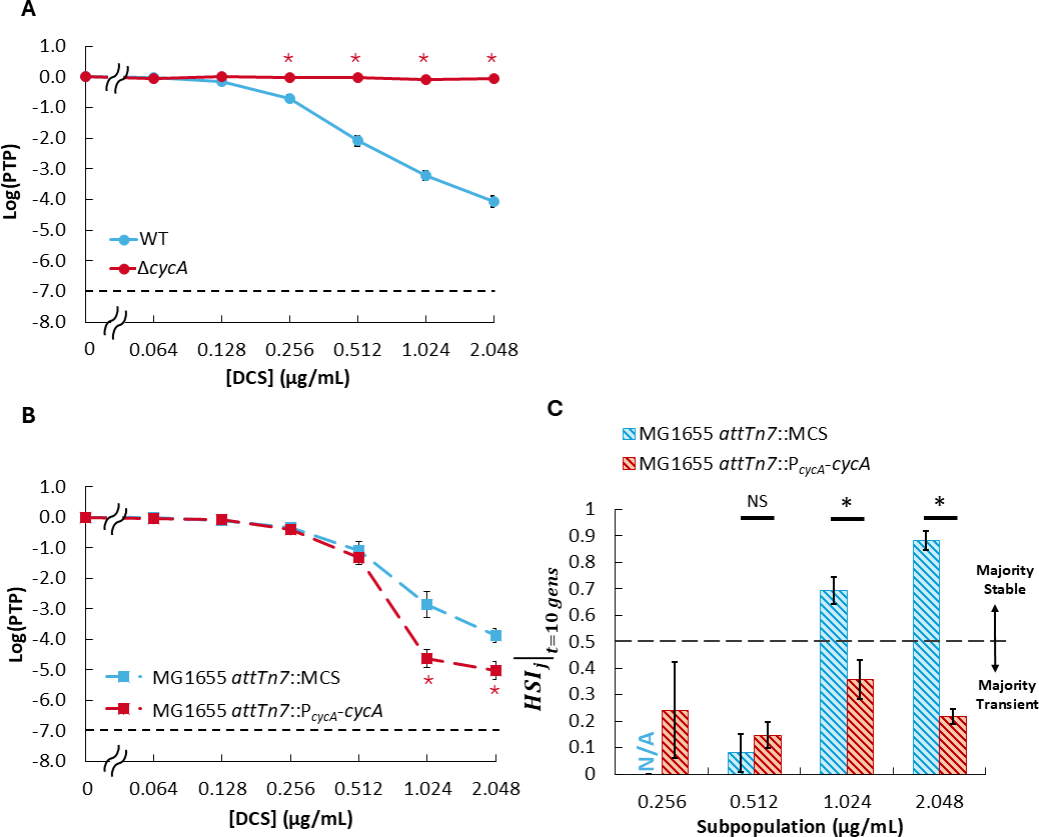
Impact of *cycA* gene dosage on DCS heteroresistance and subpopulation stability for MG1655. (**A**) PAP of Δ*cycA* compared to wild type (WT). (**B**) PAP and (**C**) ${HSI}_{j}{|}_{t=10\;gens}$ of strain harboring an additional genetic copy of *cycA* (2 total) compared to control (1 total). Data represent averages of at least three biological replicates, and error bars represent SEM. Dashed lines in panels **A** and **B** indicate the threshold for heteroresistance. Dashed line in panel **C** delineates the point of transition between subpopulation stability states (majority transient for ${HSI}_{j}{|}_{t}<0.5$ vs majority stable for ${HSI}_{j}{|}_{t}>0.5$). N/A indicates concentration where subpopulation stability was not calculated due to functional limitations of heteroresistance stability index (HSI; 0× HNIC control subpopulations did not produce minority subpopulations after propagation during subpopulation stability PAP, ${PTP}_{j}^{0}{|}_{t}>0.5$, for the majority of replicates). Star represents statistical significance by one-way ANOVA and Tukey *post hoc* analysis (*P* < 0.05). NS = no significance.

Consistent with our expectations, Δ*cycA* exhibited homogeneous resistance up to at least 2.048 µg/mL (HNIC_DCS_ ≥ 2.048 µg/mL; Fig. 3A) and MG1655 *attTn7*::P*_cycA_-cycA* exhibited increased sensitivity to DCS at and above 1.024 µg/mL compared to a control harboring the non-protein coding multiple cloning sequence (MCS) at *attTn7* (MG1655 *attTn7*::MCS; Fig. 3B). Because deletion of *cycA* resulted in homogeneous resistance, and our stability metric (${HSI}_{j}{|}_{t}$) only applies for minority subpopulations above the HNIC, we were unable to assess subpopulation stability for Δ*cycA* at the concentrations considered here. For the strain with two genetic copies of *cycA*, we observed decreased subpopulation stability compared to control, as indicated by significantly lower ${HSI}_{j}{|}_{t=10\;gens}$ measurements at 1.024 µg/mL and 2.048 µg/mL DCS, without significant impacts on the subpopulation stability at lower concentrations (Fig. 3C; Fig. S2). Collectively, these results suggest that even modest changes in *cycA* dosage can substantially impact both DCS heteroresistance and subpopulation stability.

### D-alanine ligase DdlB contributes to the transience of DCS heteroresistance

**Fig 4:**
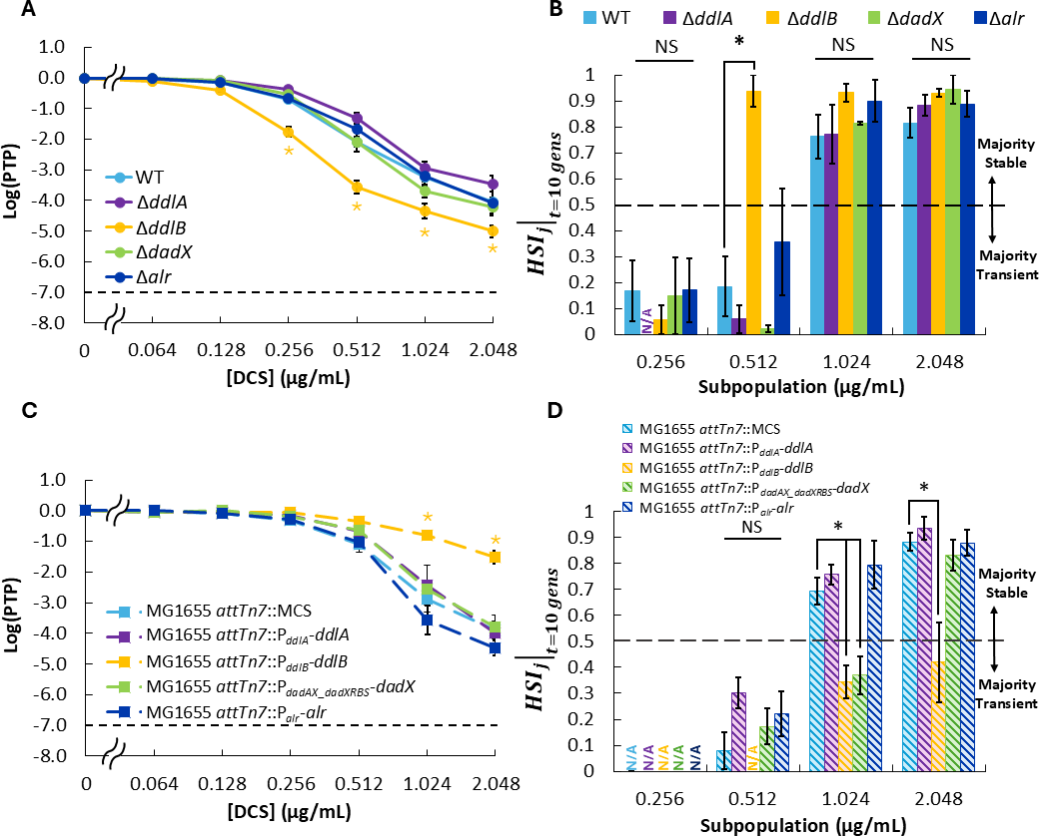
Impact of DCS target gene dosage on heteroresistance and subpopulation stability for MG1655. (**A**) PAP and (**B**) ${HSI}_{j}{|}_{t=10\;gens}$ of single-deletion mutants compared to WT. (**C**) PAP and (**D**) ${HSI}_{j}{|}_{t=10\;gens}$ of strains harboring additional copies of DCS target genes relative to control (2 compared to 1). Data represent averages of at least three biological replicates, and error bars represent SEM. Dashed lines in panels **A** and **C** indicate the threshold for heteroresistance. Dashed line in panels **B** and **D** delineates the point of transition between subpopulation stability states (majority transient for ${HSI}_{j}{|}_{t}<0.5$ vs majority stable for ${HSI}_{j}{|}_{t}>0.5$). N/A indicates concentration where subpopulation stability was not calculated due to functional limitations of heteroresistance stability index (HSI; 0× HNIC control subpopulations did not produce minority subpopulations after propagation during subpopulation stability PAP, ${PTP}_{j}^{0}{|}_{t}>0.5$, for the majority of replicates). Star represents statistical significance by one-way ANOVA and Tukey *post hoc* analysis (*P* < 0.05). NS = no significance.

Next, we investigated how gene dosage of each of the DCS targets (*ddlA*, *ddlB*, *dadX*, and *alr*) contributes to DCS heteroresistance and subpopulation stability. First, we generated single deletion mutants of each of the genes, which we hypothesized would increase sensitivity to DCS, because higher DCS-to-target ratios would result for the remaining targets. Interestingly, we observed that Δ*ddlB* was the only deletion mutant that displayed increased sensitivity to DCS (Fig. 4A), with subpopulation frequencies at and above 0.256 µg/mL DCS being significantly lower for Δ*ddlB* than those of wild-type (WT). Furthermore, when we measured the subpopulation stabilities of our deletion strains, we observed that Δ*ddlB* displayed higher stability at lower concentrations compared to WT. Specifically, the measured ${HSI}_{j}{|}_{t=10\;gens}$ of subpopulations harvested at 0.512 µg/mL DCS were significantly higher for Δ*ddlB* than those of WT, whereas there was no significant difference in ${HSI}_{j}{|}_{t=10\;gens}$ measurements between Δ*ddlA*, Δ*dadX*, Δ*alr*, and WT at any DCS concentration (Fig. 4B; Fig. S3). This finding contrasts with the case of enhanced DCS susceptibility conferred by an additional copy of *cycA* (Fig. 3B and C), where the window of transience expanded to higher DCS concentrations; here, increased DCS susceptibility produced an expansion of stable heteroresistance to lower DCS concentrations.

To determine the impact of increasing DCS target gene dosage, we generated MG1655 strains harboring an additional copy of *ddlA*, *ddlB*, *dadX*, or *alr* at *attTn7* and likewise measured the impact on PAP and ${HSI}_{j}{|}_{t=10\;gens}$. Analogous to the deletion mutants, MG1655 *attTn7*::P*_ddlB_-ddlB* was the only strain harboring two copies of a DCS target that exhibited significantly decreased sensitivity to DCS relative to the control (MG1655 *attTn7*::MCS), with the HNIC for MG1655 *attTn7*::P*_ddlB_-ddlB* (HNIC_DCS_ = 0.512 µg/mL) and subpopulation frequency at and above 1.024 µg/mL all being higher than that of MG1655 *attTn7:*:MCS (HNIC_DCS_ = 0.256 µg/mL; Fig. 4C). This decreased sensitivity also conferred decreased stability of the 1.024 and 2.048 µg/mL subpopulations compared to control, a trend that mirrors our observations that increasing sensitivity through *ddlB* deletion conferred increased stability (Fig. 4D; Fig. S4B). Interestingly, harboring an additional copy of *dadX* also decreased the stability of the subpopulation harvested at 1.024 µg/mL DCS compared to the control (Fig. 4D; Fig. S4C), despite the minimal impact of *dadX* gene dosage variation on DCS sensitivity (Fig. 4A and C). Taken together, these results suggest that gene dosage of *ddlB*, and to a lesser extent *dadX*, can influence the heteroresistance and stability of MG1655 to DCS.

### Removal of DCS target isozymes increased sensitivity and decreased heteroresistance transience

**Fig 5:**
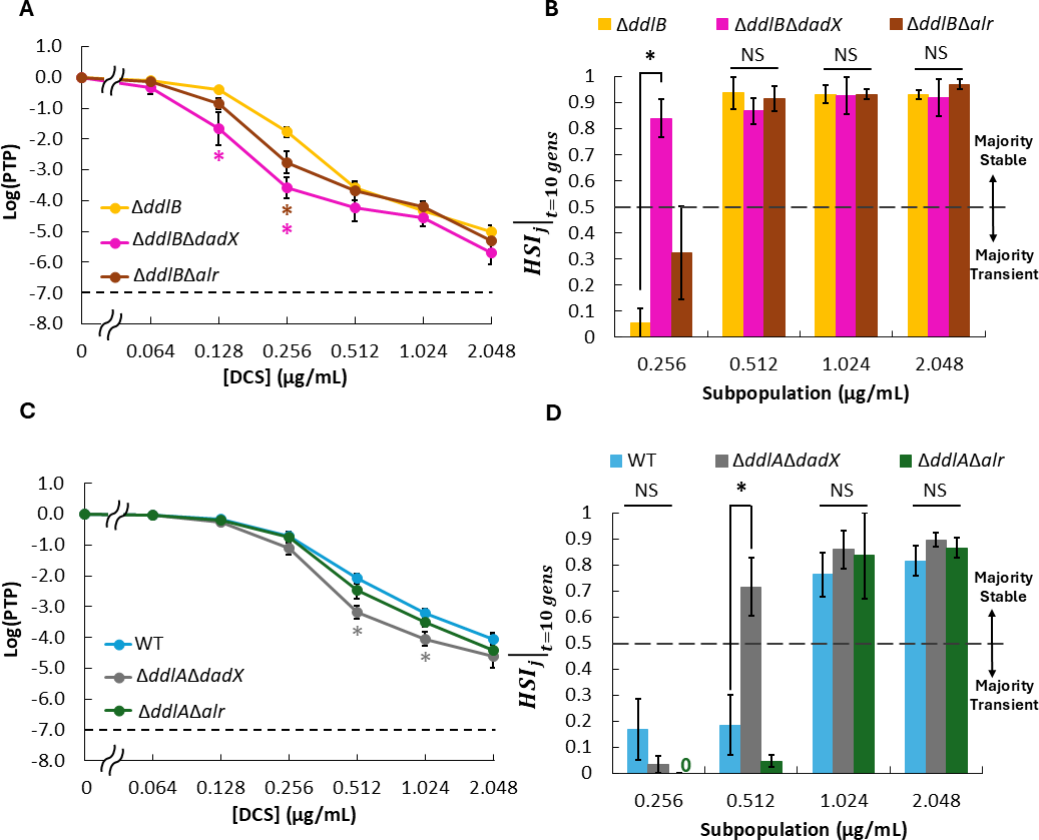
Impact of DCS target double-deletion strains on heteroresistance and subpopulation stability for MG1655. (**A**) PAP and (**B**) ${HSI}_{j}{|}_{t=10\;gens}$ of viable double-deletion mutants containing Δ*ddlB* compared to Δ*ddlB* single deletion strain. (**C**) PAP and (**D**) ${HSI}_{j}{|}_{t=10\;gens}$ of viable double-deletion mutants including Δ*ddlA* compared to WT. Data represent averages of at least three biological replicates, and error bars represent SEM. Dashed lines in panels **A** and **C** indicate the threshold for heteroresistance. Dashed line in panels **B** and **D** delineates the point of transition between subpopulation stability states (majority transient for ${HSI}_{j}{|}_{t}<0.5$ vs majority stable for ${HSI}_{j}{|}_{t}>0.5$). Star represents statistical significance by one-way ANOVA and Tukey *post hoc* analysis (*P* < 0.05). NS = no significance.

Given the presence of two isozymes for D-alanine ligase and D-alanine racemase (59), we investigated how DCS heteroresistance and stability compared to when only one isozyme for each enzymatic function was available. To accomplish that, we generated all viable double-deletion mutants of the four genes (Δ*ddlB*Δ*dadX*, Δ*ddlB*Δ*alr*, Δ*ddlA*Δ*dadX*, and Δ*ddlA*Δ*alr*) and measured the impact on PAP (Fig. 5). We observed that deletion of *dadX* or *alr* in Δ*ddlB* further increased sensitivity to DCS, with the subpopulation frequency of Δ*ddlB*Δ*dadX* being significantly lower than Δ*ddlB* at 0.128 µg/mL and 0.256 µg/mL, and that of Δ*ddlB*Δ*alr* being significantly lower at 0.256 µg/mL (Fig. 5A). However, the increased sensitivity only impacted the subpopulation stabilities of Δ*ddlB*Δ*dadX*, which showed a higher ${HSI}_{j}{|}_{t=10\;gens}$ compared to Δ*ddlB* at 0.256 µg/mL DCS, whereas the subpopulation stabilities of Δ*ddlB*Δ*alr* did not significantly differ from Δ*ddlB* at any concentration (Fig. 5B; Fig. S5A and B). Similarly, concurrent deletion of *dadX* and *ddlA* increased sensitivity to DCS (as seen by a significant drop in subpopulation frequency at 0.512 µg/mL and 1.024 µg/mL for Δ*ddlA*Δ*dadX* compared to WT; Fig. 5C), which resulted in significantly higher ${HSI}_{j}{|}_{t=10\;gens}$ at 0.512 µg/mL compared to WT (Fig. 5D; Fig. S5C). On the other hand, deletion of both *alr* and *ddlA* did not substantially alter DCS sensitivity (Fig. 5C) or subpopulation stability compared to WT (Fig. 5D; Fig. S5D). We note that comparisons for double deletion mutants not involving Δ*ddlB* were conducted with WT because none of the *ddlA*, *dadX*, or *alr* individual deletion strains deviated from WT significantly (Fig. 4A and B). These findings suggest that strains with only a single D-alanine racemase and/or D-alanine ligase could be more likely to exhibit stable heteroresistance at lower DCS concentrations. Notably, mycobacteria (59–61) and some Gram-positive bacteria (59, 60, 62) have been shown to harbor single isozymes of one or both enzymes.

### Impact of *cycA* and *ddlB* gene dosage on heteroresistance stability translates to UPEC UTI89

**Fig 6:**
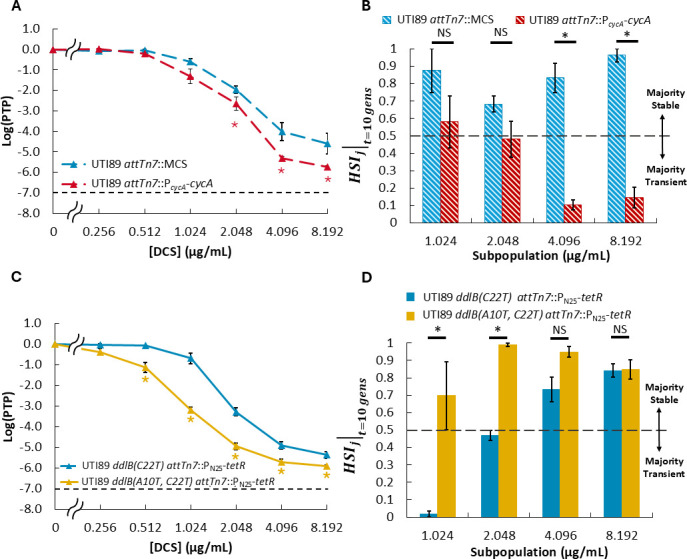
Evaluation of the impact of *cycA* and *ddlB* gene dosage on DCS heteroresistance and subpopulation stability in UTI89. (**A**) PAP and (**B**) ${HSI}_{j}{|}_{t=10\;gens}$ of strain harboring an additional genetic copy of *cycA* (2 total) compared to control (1 total). (**C**) PAP and (**D**) ${HSI}_{j}{|}_{t=10\;gens}$ of strain with premature STOP codon in *ddlB* (UTI89 *ddlB[A10T, C22T] attTn7*::P_N25_-*tetR*) compared to control (UTI89 *ddlB[C22T] attTn7*::P_N25_-*tetR*). Data represent averages of at least three biological replicates, and error bars represent SEM. Dashed lines in panels **A** and **C** indicate the threshold for heteroresistance. Dashed line in panels **B** and **D** delineates the point of transition between subpopulation stability states (majority transient for ${HSI}_{j}{|}_{t}<0.5$ vs majority stable for ${HSI}_{j}{|}_{t}>0.5$). Star represents statistical significance by one-way ANOVA and Tukey *post hoc* analysis (*P* < 0.05). NS = no significance.

To verify whether insights gleaned from heteroresistance investigations using our model strain translate to pathogenic isolates, we sought to determine whether altering DCS sensitivity of UTI89 via gene dosage alterations of *cycA* or *ddlB* had the same disparate effect on subpopulation stability as they did in MG1655. To do so, we constructed a UTI89 derivative with P*_cycA_-cycA* inserted at the Tn7 attachment site (UTI89 *attTn7*::P*_cycA_-cycA*), a strain that contains a premature STOP at the fourth codon of *ddlB* (UTI89 *ddlB[A10T, C22T] attTn7*::P_N25_-*tetR*), and their respective controls (see “Materials and Methods”). As we observed with MG1655, the UTI89 derivative harboring an additional copy of *cycA* exhibited increased sensitivity to DCS compared to its control (UTI89 *attTn7*::MCS), as indicated by significantly lower subpopulation frequencies at and above 2.048 µg/mL DCS; as well as significantly decreased stability at and above 4.096 µg/mL DCS relative to its control (Fig. 6A and B; Fig. S6A and B). In addition, our derivative harboring a premature STOP codon in *ddlB* exhibited increased sensitivity relative to its control (UTI89 *ddlB[C22T] attTn7*::P_N25_-*tetR*) at and above 0.512 µg/mL DCS, and the stability of the subpopulations harvested at and above 1.024 µg/mL DCS exhibited similarly high or increased stability compared to control (Fig. 6C and D; Fig. S6C and D). Collectively, these results reiterate the disparate impact of increasing DCS sensitivity via altered *cycA* or *ddlB* gene dosage on heteroresistance stability that was observed for MG1655, which suggests that insights obtained from heteroresistance investigations with MG1655 could be applicable to pathogenic isolates.

### Quantification of *cycA* and *ddlB* expression in gene dosage mutants

**Fig 7:**
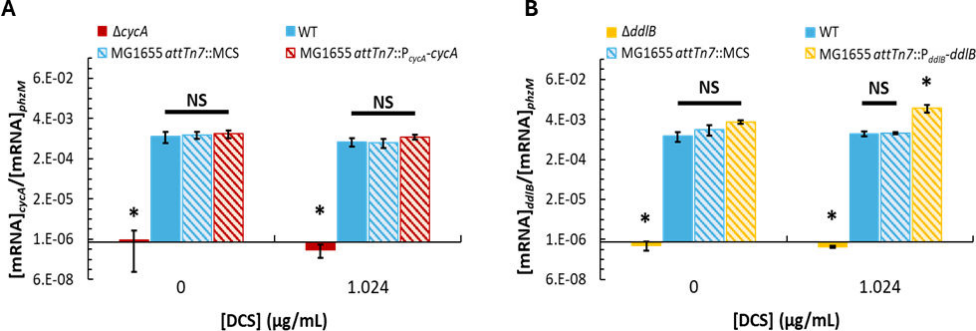
Quantifying *cycA* and *ddlB* expression levels in strains harboring 0, 1, or 2 chromosomal copies of each gene. Transcript levels of (**A**) *cycA* and (**B**) *ddlB* measured relative to *phzM* external control standard by reverse transcription-qPCR (RT-qPCR). Star represents statistical significance by one-way ANOVA and Tukey *post hoc* analysis on log-transformed data (*P* < 0.05). NS = no significance. For statistical analysis of log-transformed data, one replicate of Δ*cycA* harvested at 0 µg/mL was omitted, as the measured amplification of *cycA* in the RT^−^ control reaction was greater than in the RT^+^ reaction, resulting in a negative calculated ratio that could not be log-transformed. We note that amplification of *cycA* in samples from Δ*cycA* (sample omitted) was expected to be negligible.

While the above analyses focused on mutants with discrete numbers (0, 1, and 2) of genomic loci for specific genes, the extent to which expression of those genes varied between mutants was unknown. Considering that differences in gene expression could impact heteroresistance, we quantified *cycA* and *ddlB* expression in their gene dosage mutants and controls in untreated and heteroresistant subpopulations using quantitative PCR (qPCR). In untreated cultures, expression of *cycA* was comparable between 1- and 2-copy strains, whereas the deletion mutant had negligible amplification (Fig. 7A). In heteroresistant subpopulations that had grown on 1.024 µg/mL DCS, the same expression pattern was observed (Fig. 7A). These data show that addition of a second *cycA* copy expressed from its native promoter at the *attTn7* site did not lead to appreciable differences in *cycA* expression, even though that mutant did exhibit lower heteroresistance to DCS and enhanced transience (Fig. 3B and C).

For *ddlB*, insignificant differences in expression were observed between 1- and 2-copy strains in untreated populations, whereas the deletion mutant had negligible amplification (Fig. 7B). However, in heteroresistant subpopulations that had grown on 1.024 µg/mL DCS, the deletion mutant had negligible amplification, the 1-copy strains exhibited similar expression, and the 2-copy strain had significantly higher *ddlB* expression than other strains. Specifically, the heteroresistant subpopulation of the 2-copy mutant expressed approximately sixfold more *ddlB* transcripts than those of the 1-copy controls (Fig. 7B). These data suggest that higher *ddlB* expression was selected for in the 2-copy mutant in the presence of 1.024 µg/mL DCS, which is consistent with the higher heteroresistance exhibited by that strain (Fig. 4C). Interestingly, the heteroresistant subpopulation of that strain was more transient than its 1-copy counterpart (Fig. 4D), which suggests that the resistance phenotype of the 2-copy mutant was less advantageous in DCS-free media. Collectively, these qPCR results reveal that gene copy variations that impact heteroresistance and its stability are not always associated with gene expression differences, although instances of gene expression correlating with copy number variations can occur.

## DISCUSSION

With high global rates of AMR being reported for a number of antibiotic-microbe combinations under surveillance by the World Health Organization (2), it is imperative that we better understand how bacteria evade the effects of antibiotic treatment (2–9). Heteroresistance is an underappreciated phenomenon that has been suggested to be a significant contributor to clinical treatment failure and the evolution of AMR (10, 14). Knowledge of the factors that contribute to the incidence and stability of heteroresistance will not only improve understanding of why treatments fail but also provide clues to prevent the development of wholly resistant infections from heteroresistant subpopulations.

One mechanism through which transient heteroresistance can occur involves the chromosomal or plasmid-mediated increase of AMR gene dosage in resistant subpopulations (18, 37, 44, 45). Although initial studies associating increased AMR gene copy number with transient heteroresistance observed such increases to occur via tandem gene amplifications (18), more recent studies have observed that gene copy number variation can also arise through transposition of AMR genes onto plasmids and/or variation in plasmid copy number (37). For example, Nicoloff and colleagues reported that transiently resistant subpopulations of a *K. pneumoniae* clinical isolate, which was heteroresistant to multiple antibiotics and harbored multiple plasmids, exhibited increased copy numbers of AMR genes that were carried on plasmids (37). Harboring increased AMR gene copies negatively impacted growth in the absence of antibiotics, resulting in decreases in AMR gene copy number and concomitant decreases in MIC after treatment (37). Pal and Andersson similarly reported that increases in AMR gene dosage acquired during antibiotic treatment negatively impacted subpopulation fitness in antibiotic-free media for four Gram-negative clinical isolates transiently heteroresistant to gentamicin, tobramycin, or tetracycline (45). These recent studies illustrate how gene dosage variation can arise through a variety of mechanisms and underscore how gene dosage has emerged as an important factor that influences heteroresistance and its stability for a variety of species and antibiotics.

Motivated by accumulating evidence for gene dosage as a driver of heteroresistance, we chose to explore how the dosage of genes within an antibiotic’s target network impacts heteroresistance and its stability. To accomplish that, we sought to use MG1655 as a genetically tractable model organism to precisely modulate chromosomal gene dosage through genetic deletion or insertion of an additional gene copy at the Tn7 attachment site. Importantly, spontaneous loss or duplication events, even at distant loci (63), are observed frequently in bacteria and are understood to play an important role in evolution (64, 65), underscoring the biological relevance of this approach. However, we first had to determine whether MG1655 exhibited heteroresistance, and we also sought to assess whether what might be learned with MG1655 could apply to pathogenic strains. We found that MG1655 exhibited heteroresistance to FOS, TOB, and DCS, which closely resembled that of UPEC strain UTI89 (Fig. 1A and B). Furthermore, we found that the DCS targets whose gene dosages impacted heteroresistance and its stability in MG1655 (Fig. 3B and C, 4A and B) also impacted DCS heteroresistance of UTI89 and its stability in a similar fashion (Fig. 6), despite the fact that MG1655 was more sensitive to DCS (MIC_DCS_ = 0.256 µg/mL; Fig. S8A) than UTI89 (MIC_DCS_ = 1.024 µg/mL; Fig. S8B). These findings highlight the utility of MG1655 as a model organism for heteroresistance research whose results translate well to UPEC strains. However, the applicability of these trends to other pathogens (e.g., *Mycobacterium tuberculosis* or multidrug-resistant isolates) will need to be assessed with future research.

Regarding heteroresistance stability, the rate and/or degree of subpopulation reversion is an important feature for understanding and characterizing heteroresistance (12, 66). To provide a quantitative measure of heteroresistance stability, we developed a framework (subpopulation stability assay and ${HSI}_{j}{|}_{t}$ metric [equation 1; Fig. 2B]) with which the stability or transience of heteroresistance can be quantified on a subpopulation-wide scale. That framework can be applied for any bacterial species that produces countable colonies on media agar containing an antibiotic of interest, and the ${HSI}_{j}{|}_{t}$ provides a ready estimate of the proportion of a subpopulation that remains resistant to that antibiotic after treatment concludes without the stochastic noise associated with estimating that quantity from single-colony measurements. Consequently, ${HSI}_{j}{|}_{t}$ is well suited for quantitative assessments of stability in heteroresistant subpopulations that are not wholly transient nor wholly stable, which are encountered frequently. However, it is worth noting that ${HSI}_{j}{|}_{t}$ should not be used when the control subpopulation (harvested from plates containing 0 µg/mL DCS) fails to produce a minority subpopulation at a given concentration during the subpopulation stability assay because the denominator approaches zero, which occurs at low DCS concentrations near the HNIC. Additionally, the HSI framework, which requires multiple antibiotic-containing plates to isolate subpopulations and an additional period of overnight growth to assess subpopulation stability, is more time and resource intensive than routine clinical susceptibility tests (disc diffusion, *E*-test, or broth microdilution), which could make its integration into routine clinical diagnostics more challenging.

Using MG1655 and ${HSI}_{j}{|}_{t}$ to explore how copy number of DCS network nodes (i.e., transporter and drug targets) influence heteroresistance and its stability, we observed that chromosomally altering the gene dosage of *cycA* or *ddlB* had a substantial effect on the sensitivity of MG1655 to DCS (Fig. 3A and B and 4A and C). Those results underscore that *cycA* copy number is a limiting factor for DCS activity, as even an increase in copy number from one to two increases the inhibitory effect of DCS. Furthermore, these findings indicate that gene dosage variation of each DCS target does not impact DCS sensitivity to equal degrees, as DdlB was the only DCS target whose individual variation in gene dosage impacted DCS heteroresistance here (Fig. 4A and C). Interestingly, Suarez and Martiny reported that increasing the copy number of *ddlA*, which encodes an isozyme of DdlB, with a high copy plasmid conferred resistance to DCS using an *E. coli* cloning strain (67). Here, individual variation of *ddlA*, which included zero, one, or two chromosomal copies, did not alter sensitivity of MG1655 to DCS (Fig. 4A and C). However, deletion of *ddlA* did impact DCS sensitivity when it was combined with Δ*dadX* (Fig. 5C), and a similar increase in sensitivity was observed when *dadX* was deleted along with *ddlB* (Fig. 5A). The fact that only a subset of DCS targets exhibited gene dosage effects on DCS heteroresistance could be a result of differences in the DCS affinities of target enzymes and/or variations in their regulation/expression. Reports by Lambert and Nauhaus (68) and Zawadzke and colleagues (39) suggest that *E. coli* D-alanine ligases (DdlA and DdlB) could have over 10-fold higher affinity for DCS than do D-alanine racemases (DadX and Alr), and the DCS affinity of DdlA is approximately threefold higher than DdlB (39). Those reports indicate that DdlA would be inhibited at lower DCS concentrations than the other targets, so deletion of its “backup” isozyme DdlB would result in the greatest impact on DCS sensitivity. Furthermore, Wild and colleagues reported that DadX expression accounted for a larger proportion of D-alanine racemase activity in K12 strains than did Alr (40), which could explain why double-deletion mutants involving Δ*dadX* exhibited a greater increase in DCS sensitivity than those involving Δ*alr* (40).

While the impacts of transporter and target gene dosage variations on DCS sensitivity aligned with our expectations, their divergent impact on subpopulation stability was not anticipated. Conceivably, a cell that belongs to a resistant subpopulation (and thus able to form a colony during treatment) would be in one of three states: (i) the cell possesses or acquires a resistance mutation that is not detrimental to cell fitness in the absence of antibiotic; (ii) the cell possesses or acquires a resistance mutation that is detrimental to cell fitness in the absence of antibiotic; or (iii) the cell is in or enters into a temporary phenotypic state that allows for growth and that is selectively maintained during treatment. If a subpopulation is dominated by cells in state (i), then the subpopulation should be measured to be majority stable by ${HSI}_{j}{|}_{t}$, whereas subpopulations which are dominated by cells in states (ii) and/or (iii) would be expected to exhibit majority transience due to compensatory mutations and/or reversion to the cell state prior to antibiotic exposure. Thus, when *cycA* or *ddlB* gene dosage is increased, the concomitant increase in transience exhibited by DCS heteroresistant subpopulations harvested at 1.024 µg/mL and 2.048 µg/mL (Fig. 3C and 4D) suggests that there are more cells in states (ii) and/or (iii) compared to their respective controls at those concentrations. In the case of increased *cycA* gene dosage expanding transience to higher DCS concentrations without changing *cycA* expression on a subpopulation-wide level (Fig. 7A), one potential path could be a mutation to DdlA because it has the highest DCS-binding affinity and is the main D-alanine ligase, and thereby, a mutation to it would likely impact fitness. In the case of increasing *ddlB* dosage, qPCR results suggest that increased expression of *ddlB* could be one mechanism through which transient heteroresistance to DCS arises in strains harboring two chromosomal copies of *ddlB* (Fig. 7B); however, whether that increased *ddlB* expression arises by phenotypic or genetic means remains to be elucidated. Conversely, when *ddlB* gene dosage is reduced, the expansion of stability to lower DCS concentrations suggests that there are more cells in state (i) compared to the control at those concentrations. One potential path could be through loss-of-function mutations in *cycA* because *cycA* is non-essential for growth in the rich media used for propagation (46, 69), and the amino acid substrates of *cycA* are not present in the PAP media used here. In any case, it is apparent that there is a diversity of mechanisms occurring that confer resistance to cells within each subpopulation because those subpopulations often do not exhibit complete transience or complete stability (Fig. S2 to S4).

Overall, while the results presented here focus on the impact of the DCS target network on heteroresistance, we anticipate that the trends observed will be applicable to other antibiotics as well. Other than DCS, there are several additional antibiotic classes that target multiple enzymes, which include penicillins, cephalosporins, and carbapenems that all target penicillin-binding proteins (57, 70), and FQs that target both DNA gyrase and topoisomerase IV (53). The data presented here suggest that strains are more likely to exhibit transient heteroresistance if they have a higher number of genetically independent enzymes inhibited by that antibiotic. Additionally, there are several other antibiotics that enter through one or multiple transporters, including FOS, which enters the cytoplasm through inner membrane transporters UhpT and GlpT (42), and cefiderocol, a cephalosporin that enters through outer membrane porins (56) as well as various iron transporters (54, 55). Interestingly, cefiderocol heteroresistance has been suggested as a potential cause of treatment failure of seemingly susceptible infections caused by *Acinetobacter baumannii* and other *Acinetobacter* species (71–73). One phase 3 clinical trial found that cefiderocol treatment of carbapenem-resistant *Acinetobacter* infections resulted in higher all-cause mortality rates compared to treatment with best available therapy, despite post-treatment MICs of all cefiderocol-treated isolates being measured to be susceptible (74). Notably, several iron-uptake systems have been identified in *A. baumannii* (75), and more than one iron uptake gene (*piuA* and *pirA*) has been associated with cefiderocol uptake by this species (76). While the applicability of the observed trends to other antibiotics/classes is subject to the kinetics, affinities, and overall abundances of each target network component and its potential mutations, the data presented here suggest that it may be advantageous to increase both the number of targets and the number of importers for antibiotics so as to not only increase the potency of a drug but also to minimize the development of stable resistance. One way this could be accomplished is through the design of dual-mechanism antibiotics, such as irresistin (77) that disrupts both folate synthesis (via inhibition of dihydrofolate reductase) and membrane integrity (via its isopropylbenzene moiety) (77), where the latter feature could serve a role as both a secondary target and mechanism of increased intracellular transport.

## MATERIALS AND METHODS

### Media and reagents

All media components and reagents used in this study are listed in Table S1. For details regarding preparation of media and all media supplements, see Supplemental methods.

### Plasmid and strain construction

All plasmids used for this study are listed in Table S2. All plasmids were constructed via Gibson assembly (using the NEB Gibson Assembly Master Mix) or site-directed mutagenesis (using the NEB Q5 Site-Directed Mutagenesis Kit). Template DNA for all cloning steps was extracted using the QIAGEN QIAprep Spin Miniprep kit (for plasmid templates) or the QIAGEN Dneasy Blood and Tissue Kit (for genomic DNA [gDNA] templates) and eluted in autoclaved water. For all Gibson assemblies, cloning steps were conducted using Phusion High Fidelity DNA Polymerase, and PCR-amplified fragments for Gibson assembly and Sanger sequencing were gel-extracted beforehand using QIAGEN Qiaquick Gel Extraction Kits. All plasmid sequences were confirmed by PCR, Sanger sequencing, and/or whole plasmid sequencing by Plasmidsaurus.

All strains in this study are derivatives of *E. coli* K-12 MG1655 or UPEC UTI89 and are listed in Table S3. All strains harboring an additional copy of DCS target network components or non-protein coding MCS control were generated using Tn7 transposition via transformation with pGRG25 or its derivatives (Table S2) as described by McKenzie and Craig (78). MG1655 genetic deletion mutants were generated using P1 bacteriophage transduction from the Keio collection, and kanamycin resistance cassettes (*kanR*) were cured by FLP recombination via transformation with pCP20 (47, 48). UTI89 *ddlB(A10T, C22T) attTn7*::P_N25_-*tetR* and its control UTI89 *ddlB(C22T) attTn7*::P_N25_-*tetR* were generated by first inserting P_N25_-*tetR* at *attTn7* by Tn7 transposition (78), followed by CRISPR Optimized MAGE Recombineering as described by Ronda and colleagues (49) using pMAZSK gRNA*_ddlB_* (Table S2) and the following mutagenic oligos (bases in uppercase/bold indicate STOP and protospacer-adjacent motif [PAM] mutations): 5′-gaggaagaacaacatgactgatTaaatcgcggtcTtgttgggcgggacctccgctgagcgggaagtttct-3′ (STOP and PAM mutation) and 5′-gaggaagaacaacatgactgataaaatcgcggtcTtgttgggcgggacctccgctgagcgggaagtttct-3′ (PAM mutation only).

We note that *ddlB* disruption via insertion of *kanR* was attempted via the method of Datsenko and Wanner (48), but we were unable to obtain a successful knockout mutant, potentially due to polar effects of disrupting the *ddlB* coding sequence on flanking essential genes (*murC* and *ftsQ*). For that reason, we elected to mutate the fourth codon of *ddlB* to a STOP codon instead, which was successful. Intended modifications of all strains were verified by PCR and/or Sanger sequencing (Tables S2 to S4). For additional details of strain constructions, see Supplemental methods.

### Population analysis profiling

To conduct PAP on parental strains, strains were inoculated into 2 mL LB liquid media from −80°C, 25% glycerol stocks generated from single colonies in sterile glass test tubes, and grown at 37°C while shaking overnight for 16 hours. After overnight growth, 1 mL of each culture was pelleted at 15,000 rpm for 3 minutes in a tabletop microcentrifuge, the entire supernatant was removed, and cell pellets were resuspended in 0.85% NaCl. Fivefold serial dilutions were prepared in 0.85% NaCl. Ten microliter spots of undiluted culture and fivefold dilutions were plated on antibiotic-free agar media or agar media supplemented with antibiotic at 0.5×, 1×, 2×, 4×, 8×, and 16× of the HNIC of antibiotic measured for MG1655 (for MG1655 and its derivatives) or UTI89 (for UTI89 and its derivatives). HNICs were determined to be the highest drug concentrations at which log-transformed proportion of the total population (PTP) measurements were not significantly different from 0 (*P* > 0.05 by one-way ANOVA with Tukey post-hoc analysis) and the majority of the cultures grew (PTP_1× HNIC_ ≥ 0.5). HNICs for all strains can be found in Table S5. For antibiotics with no known transporters (CIP and TOB), cation-adjusted Mueller-Hinton agar (CAMHA) was used. For antibiotics with known transporters (FOS and DCS), an appropriate medium was selected to ensure expression of the transporter. For FOS, CAMHA was supplemented with 25 µg/mL glucose-6-phosphate. For DCS, M9 + 10 mM glucose + 1.5% agar was used. Due to nicotinamide (NIC) auxotrophy of UTI89 resulting from an A28V mutation in NadB (79), minimal media agar plates were also supplemented with 1 µg/mL NIC for assays with UTI89 and its derivatives. We confirmed that the addition of NIC to minimal media plates did not appreciably impact the DCS sensitivity of WT MG1655 (Fig. S7A). After 16 hours (for CIP, TOB, and FOS on complex media agar) or 40 hours (for DCS on minimal media agar) of incubation at 37°C, CFUs were enumerated, and the PTP growing at each antibiotic concentration was calculated by


(2) $$PTP_{j}=\frac{{\left(CFU\;count\right)}_{j}\;x\;DF_{j}}{{\left(CFU\;count\right)}_{\;0xHNIC}\;x\;DF_{0xHNIC}},$$

where *j* indicates the antibiotic concentration, and DF indicates the dilution factor of the spot where CFUs were counted

### DCS heteroresistant subpopulation stability assay

To isolate DCS-resistant minority subpopulations for harvesting and propagation, parent strains were inoculated into 2 mL LB liquid media from a −80°C, 25% glycerol stock in a glass test tube and grown at 37°C while shaking overnight for 16 hours. Following overnight growth, 1–2 mL of culture was pelleted at 15,000 rpm for 3 minutes, the supernatant was removed, and pellets were resuspended in 0.85% NaCl. One hundred microliter of appropriate dilutions prepared in 0.85% NaCl was then spread onto M9 + 10 mM glucose + 1.5% agar plates containing no antibiotic or twofold increasing concentrations of DCS to yield ~300 to ~1 × 10^3^ CFUs per concentration. For MG1655 and its derivatives, DCS plates were prepared at 0.256 µg/mL (2× HNIC_DCS_) up to 2.048 µg/mL (16× HNIC_DCS_). For UTI89 and its derivatives, DCS plates were prepared at 1.024 µg/mL (2× HNIC_DCS_) up to 8.192 µg/mL (16× HNIC_DCS_). As done for PAP assays, minimal media agar plates were additionally supplemented with 1 µg/mL NIC for assays with UTI89 and its derivatives. We confirmed that the addition of NIC to minimal media plates did not significantly impact the DCS subpopulation stability of WT MG1655 (Fig. S7B and C). After incubating for 40 hours at 37°C, CFUs were harvested at each concentration by pipetting ~1 mL of sterile, drug-free LB onto each plate and continually swirling/pipetting until the media was visibly turbid (total culture volume recovered after harvest was ~500 µL due to some media absorption by the plates). The harvested subpopulations were then washed once to remove any residual antibiotic by centrifuging in a tabletop centrifuge at 15,000 rpm for 3 minutes, removing the entire supernatant, and resuspending pellets in 500 µL antibiotic-free LB. The washed cultures were diluted to an OD_600_ ~0.005 (~1/1,000 of terminal density, such that one overnight constitutes ~10 generations of propagation) in 2 mL drug-free LB in sterile glass test tubes and grown at 37°C while shaking. After 24 hours, PAP was performed on each culture as described above. For analysis of subpopulation stability beyond 10 generations, cultures were also diluted 10^3^-fold by adding 2 µL of each overnight culture into 2 mL fresh, drug-free LB in sterile glass test tubes and returned to a 37°C shaker. This was repeated every 24 hours for up to five overnights, and PAP was performed after overnights 1, 3, and 5 (corresponding to propagation for ~10 generations, ~30 generations, and ~50 generations, respectively).

### Derivation of HSI

To arrive at the quantitative framework for calculating the proportion of a heteroresistant subpopulation that maintains its elevated resistance (i.e., is stable) after treatment, we first considered the fraction of a population resistant to antibiotic concentration *j* during an initial PAP and assumed that those cells resistant to concentration *j* can be either stable or transient:


(3) $${{PTP}_{j}|}_{t_{i}}=\frac{{N_{j}|}_{t_{i}}}{{N_{0}|}_{t_{i}}}$$

(4) $${N_{j}|}_{t_{i}}=N_{j,\text{ stable }}+N_{j,\text{ transient }}$$

$$\begin{array}{rl} \text{ subscript } & \equiv \text{ antibiotic concentration }\\ & 0\equiv \text{ untreated }(0xHNIC)\\ N & \equiv \text{ number of cells that grow }\\ t_{i} & \equiv \text{ initial PAP} \end{array}$$

After those resistant cells are harvested, propagated without antibiotic (assuming equal expansion of cells during propagation), and a second PAP is performed:


(5) $$\begin{array}{rl} {{PTP}_{j}^{j}|}_{t} & =\frac{{N_{j}^{j}|}_{t}}{{N_{0}^{j}|}_{t}}\\ & =\underset{\begin{array}{c} ↓\\ \text{Fraction of subpop. }j\text{ from }t_{i}\text{ that are}\\ \text{stable after propagation (defined as }{HSI_{j}|}_{t}\text{)} \end{array}}{\underbrace{\frac{N_{j,stable}}{{N_{j}|}_{t_{i}}}}}\times \underset{\begin{array}{c} ↓\\ \text{Fraction of stably resistant cells that can grow}\\ \text{during second PAP (assumed to be 1)} \end{array}}{\underbrace{{PTP}_{j}^{stable}{|}_{t}}}\\ & +\underset{\begin{array}{c} ↓\\ \text{Fraction of subpop. }j\text{ from }t_{i}\text{ that are}\\ \text{transient after propagation} \end{array}}{\underbrace{\frac{N_{j,transient}}{{N_{j}|}_{t_{i}}}}}\times \underset{\begin{array}{c} ↓\\ \text{Fraction of transiently resistant cells that can grow}\\ \text{during second PAP (assumed to be equivalent}\\ \text{to untreated control)} \end{array}}{\underbrace{{PTP}_{j}^{0}{|}_{t}}} \end{array}$$

$$\begin{array}{rl} \text{ superscript } & \equiv \text{ concentration from which resistant cells were harvested }\\ & t\equiv \text{ time propagated without antibiotic before second PAP } \end{array}$$

Rearranging equation 5:


(6) $$\frac{N_{\text{j,stable }}}{{N_{j}|}_{t_{i}}}\times {{PTP}_{j}^{\text{stable }}|}_{t}={{PTP}_{j}^{j}|}_{t}-\frac{N_{j,\text{ transient }}}{{N_{j}|}_{t_{i}}}\times {{PTP}_{j}^{0}|}_{t}$$

Note that $\frac{N_{j,\text{ stable }}}{{N_{j}|}_{t_{i}}}+\frac{N_{j,\text{ transient }}}{{N_{j}|}_{t_{i}}}=1$ from equation 4.

Substituting into equation 6:


(7) $$\frac{N_{j,\text{ stable }}}{{N_{j}|}_{t_{i}}}\times {{PTP}_{j}^{\text{stable }}|}_{t}={{PTP}_{j}^{j}|}_{t}-\left(1-\frac{N_{j,\text{ stable }}}{{N_{j}|}_{t_{i}}}\right)\times {{PTP}_{j}^{0}|}_{t}$$

Rearranging to isolate $\frac{N_{j,\text{stable}}}{{N_{j}|}_{t_{i}}}:$


(8) $$\frac{N_{j,\text{stable}}}{{N_{j}|}_{t_{i}}}\times \left({{PTP}_{j}^{\text{stable}}|}_{t}-{{PTP}_{j}^{0}|}_{t}\right)=\left({{PTP}_{j}^{j}|}_{t}-{{PTP}_{j}^{0}|}_{t}\right)$$

Rearranging and defining $\frac{N_{j,\text{ stable }}}{{N_{j}|}_{t_{i}}}$ as ${{HSI}_{j}|}_{t}$:


(9) $${{HSI}_{j}|}_{t}=\frac{{{PTP}_{j}^{j}|}_{t}-{{PTP}_{j}^{0}|}_{t}}{{{PTP}_{j}^{\text{stable }}|}_{t}-{{PTP}_{j}^{0}|}_{t}}$$

where ${PTP}_{j}^{\text{stable}}{|}_{t}$ is assumed to be 1. Thus, this equation can estimate the proportion of a given subpopulation that remains in the “resistant” state entirely from measured PAP quantities, with a value of 1 indicating all the progeny are stably resistant, whereas a value of 0 indicates all progeny have lost their elevated resistance and behave as an untreated control population. We note that, due to the functional form of equation 9, the error associated with the ${HSI}_{j}{|}_{t}$ calculation increases as ${PTP}_{j}^{0}{|}_{t}$ approaches a value of 1. Consequently, to ensure robust application of this metric, we confine this framework to evaluate the stability of subpopulations harvested from concentrations on which the control culture produces a minority subpopulation (${PTP}_{j}^{0}{|}_{t}<0.5$) for the majority of replicates.

### RT-qPCR

#### Generation of phzM RNA as external standard

External standard *phzM* RNA was prepared for qPCR as previously described (80–85). First, pET11a P_T7_-*phzM* was linearized via digestion with EcoRI-HF restriction enzyme in CutSmart buffer for 1 hour at 37°C, followed by 20 minutes at 65°C for enzyme inactivation. The resulting linear plasmid was then purified following the Qiagen PCR clean-up protocol. *phzM* RNA was then transcribed using the HiScribe T7 RNA Synthesis Kit from the linearized pET11a P_T7_-*phzM* template in 20 µL reactions. The RNA was then purified in RNase-free water following the RNeasy clean-up protocol (including on-column RNase-free DNase digestion according to the manufacturer’s protocol). Aliquots of purified RNA were stored at −80°C until RNA extraction from harvested samples could be performed.

#### Sample harvest and RNA isolation

To quantify *cycA* or *ddlB* expression levels in strains harboring 0, 1, and 2 genetic copies of *cycA* (Δ*cycA*, MG1655, MG1655 *attTn7*::MCS, and MG1655 *attTn7*::P*_cycA_-cycA*) or *ddlB* (Δ*ddlB*, MG1655, MG1655 *attTn7*::MCS, and MG1655 *attTn7*::P*_ddlB_-ddlB*), subpopulations of each strain were isolated as described in the subpopulation stability assay by plating dilutions of overnight cultures on M9 minimal media + 10 mM glucose agar plates (one without DCS, and another with 1.024 µg/mL DCS), followed by incubation at 37°C for 40 hours. Cells from those plates were then harvested using 1 mL of the same media as in the plates (M9 minimal media + 10 mM glucose ±1.024 µg/mL DCS) to maintain selective pressure, and all harvested cultures were diluted in the same media to the equivalent OD_600_ of the most dilute sample of that harvested set in 500 µL total volume (the minimum OD_600_ after this harvest ranged from 0.15 to 0.23 across all replicates). Those samples were then concentrated into 100 µL by pelleting at 15,000 rpm for 3 minutes, removing 400 µL supernatant, and resuspending pellets in the remaining supernatant. Concentrated cultures were then spread onto fresh plates of their corresponding media (M9 minimal media agar + 10 mM glucose plates ± 1.024 µg/mL DCS) and incubated at 37°C for 4 hours to allow cells to resume growth. After 4 hours, cells were again harvested with 1 mL of the same media as in plates (M9 minimal media + 10 mM glucose ± 1.024 µg/mL DCS), and 300 µL was promptly added to 600 µL RNAProtect Bacteria Reagent to attain 900 µL suspensions of cells in 2:1 RNAProtect:media. All samples were added to RNAProtect within 1 minute of removing plates from the incubator for harvesting. The OD_600_ of each harvested culture was then measured using the remaining sample, which had not been added to RNA protect (the minimum OD_600_ after the second harvest ranged from 0.1 to 0.156 across all replicates), and those measurements were used to calculate the equivalent OD_600_ of cultures in the 900 µL RNAprotect solutions. The RNAprotect cell suspensions were then diluted in 2:1 RNAProtect:media to the equivalent OD_600_ as the most dilute sample of that set (${OD}_{600}^{sample}$) in 900 µL of total volume. Those cell suspensions were then pelleted at 7,300 rpm for 10 minutes, 850 µL of the supernatant was carefully removed, and pellets were stored at −80°C until RNA extraction. The above harvest procedure was conducted a total of three times for each set of strains.

To extract RNA from all samples, pellets frozen in RNAprotect were thawed at room temperature, and 50 ng of *phzM* RNA (synthesized as described above) were added to each sample. *phzM* RNA was used as an external RNA standard for comparisons between samples. Cells were then lysed by adding 100 µL of 2 mg/mL lysozyme in Tris-EDTA buffer to each sample and incubating at room temperature, during which samples were vortexed for 10 seconds every 2 minutes for 15 minutes. Total RNA was subsequently isolated from the cell lysates using the Qiagen RNeasy Mini Kit according to the manufacturer’s instructions, including on-column digestion with RNase-free DNase, as previously described (80–85). Aliquots of purified RNA were stored at −80°C until qPCR could be performed.

#### RT-qPCR

RT-qPCR was performed as previously described (80–85). First, 50 ng of each RNA sample was converted to cDNA using TaqMan Reverse Transcription Reagents (RT^+^ samples) in 20 µL reactions according to the manufacturer’s protocol. To quantify any residual DNA (gDNA or linear pET11a P_T7_-*phzM*) remaining in each sample before reverse transcription, control reactions lacking reverse transcriptase (RT^−^ samples) were also prepared for each sample. qPCR was then conducted on each sample (RT^+^ and RT^−^) using primers that were internal to *ddlB* (for samples from strains harboring 0, 1, or 2 chromosomal copies of *ddlB*), *cycA* (for samples from strains harboring 0, 1, or 2 chromosomal copies of *cycA*), and *phzM* (all samples) on a ViiA 7 Real-Time PCR System (Thermo Fisher Scientific) in a 0.1 mL MicroAMP Fast Optical 96-well reaction plate (Thermo Fisher Scientific) for 40 amplification cycles. Each well contained 2 µL of appropriate sample (RT^+^ or RT^−^), 10 µL of Power SYBR Green qPCR Master Mix, 7 µL of nuclease-free water, and 0.5 µL each of 10 µM stocks of appropriate forward and reverse primers (see Table S4). To quantify amplification efficiency of each primer set, amplification of serial dilutions of gDNA (for *ddlB* and *cycA* primers) or linearized pET11a P_T7_-*phzM* (for *phzM* primers) was conducted on each plate to generate plots of threshold cycle (*C_T_*) vs concentration, and efficiencies were calculated from the line of best fit of those plots (linear fits used to calculate efficiency had an *R*^2^ ≥ 0.99 for all replicates). Transcript levels for genes of interest (GOI), *ddlB* or *cycA*, were then quantified relative to the external standard *phzM* according to the following formula:


(10) $${\left(\frac{n_{gene}}{n_{phzM}}\right)}_{sample}=\left[\frac{E_{phzM}^{C_{T}^{phzM,\;\;{RT}^{+}\;}}}{E_{gene}^{C_{T}^{gene,\;\;{RT}^{+}\;}}}\right]\left[\frac{1\;-E_{gene}^{\left\{C_{T}^{gene,\;\;{RT}^{+}}-C_{T}^{gene,\;\;{RT}^{-}\;}\right\}}}{1\;-E_{phzM}^{\left\{C_{T}^{phzM,\;\;{RT}^{+}}-C_{T}^{phzM,\;\;{RT}^{-}\;}\right\}}}\right]\left[\frac{{OD}_{600}^{min}}{{OD}_{600}^{sample}}\right].$$

$n_{gene}$ is the number of transcripts of the GOI in a given sample; $n_{phzM}$ is the number of *phzM* transcripts in a given sample; *E*_gene_ is the measured amplification efficiency of the primers which anneal to the GOI; *E_phzM_* is the measured amplification efficiency of the primers which anneal to *phzM*; $C_{T}^{gene,\;\;{RT}^{+}}$ and $C_{T}^{gene,\;\;{RT}^{-}}$ are the measured threshold cycles for the RT^+^ and RT^−^ reactions, respectively, when using primers that anneal to the GOI; $C_{T}^{phzM,\;\;{RT}^{+}}$ and $C_{T}^{phzM,\;\;{RT}^{-}}$ are the measured threshold cycles for the RT^+^ and RT^−^ reactions, respectively, when using primers that anneal to *phzM*; ${OD}_{600}^{sample}$ is the final OD_600_ to which the sample had been adjusted after addition to RNAprotect; and ${OD}_{600}^{min}$ is the minimum value of all of the ${OD}_{600}^{sample}$ for all three harvest replicates (${OD}_{600}^{min}=0.1$ for both *cycA* and *ddlB* strain sets; for formula derivation, see Supplemental methods).

## SUPPLEMENTAL MATERIAL

Supplemental Material mbio.01490-25-s0001.pdf Supplemental methods, figures, and tables.

ASM does not own the copyrights to Supplemental Material that may be linked to, or accessed through, an article. The authors have granted ASM a non-exclusive, world-wide license to publish the Supplemental Material files. Please contact the corresponding author directly for reuse.
