# Supplementary material for: An investigation of gene dosage reveals that increased sensitivity to D-cycloserine divergently impacts the transience of heteroresistance in Escherichia coli
Source: mBio. 2025 Aug 22;16(10):e01490-25. doi: 10.1128/mbio.01490-25 (PMC12505980; doi:10.1128/mbio.01490-25)
Supplement: Supplemental Material — Supplemental methods, figures, and tables. [file mbio.01490-25-s0001.pdf]

Supplemental methods

Media preparation

All chemicals and reagents used in this study are listed in Table S1. All solutions were prepared in autoclaved MilliQ water (resistivity = 18.2 MΩ) and sterilized with a 0.22 mm filter prior to use, with the exceptions of 5x M9 salts solution, where 64 g/L $Na_2HPO_4*7H_2O$, 15 g/L KH_2PO_4, 5 g/L NH_4Cl, and 2.5 g/L NaCl were mixed with MilliQ water and autoclaved; 0.85% NaCl, where 8.5 g/L NaCl was dissolved in MilliQ water and autoclaved; ciprofloxacin (CIP), which was prepared in 0.2N HCl before filter-sterilization; anhydrotetracycline (aTET), which was prepared in dimethyl sulfoxide; and Tris-EDTA (TE) buffer, which was prepared with 20 mL nuclease-free water, 200 µL 1M Tris-HCl (molecular biology grade, pH 8), and 40 µL 0.5 M EDTA (molecular biology grade, pH 8) to achieve a final concentration of 10 mM Tris and 1 mM EDTA.

Liquid LB media were prepared with 10 g/L tryptone, 5 g/L yeast extract, 10 g/L NaCl, and MilliQ water and autoclaved. SOB media (20 g/L tryptone, 5 g/L yeast extract, 10 mM NaCl, 2.5 mM KCl, 10 mM $MgSO_4$) was prepared by mixing tryptone and yeast extract with MilliQ water, followed by autoclaving, and adding the appropriate volumes of 5 M NaCl, 1 M KCl, and 1 M MgSO4 (all filter-sterilized) to achieve the desired final concentration. SOC media was prepared by supplementing SOB with filter-sterilized 1M glucose to a final concentration of 20 mM glucose. M9 media supplemented with 10 mM glucose was prepared by adding of the following solutions to 80 mL autoclaved water: 10 µL 1M $CaCl_2$ (filter-sterilized), 200 µL 1M $MgSO_4$ (filter-sterilized), 20 mL 5x M9 salts (autoclaved), and 1 mL 1 M glucose (filter-sterilized). The media was then filter-sterilized into a bottle that had previously been sterilized by autoclaving.

LB agar plates were prepared with 25 g/L of LB premix, 15 g/L agar, and MilliQ water and autoclaved. Cation-adjusted Mueller-Hinton agar plates were prepared with 38 g/L Mueller-Hinton II agar premix and MilliQ water and autoclaved. M9 minimal media plates (100 mL basis) were prepared by mixing 1.5 g agar with 80 mL MilliQ water, followed by autoclaving, and then addition of the following solutions: 10 µL 1M $CaCl_2$ (filter-sterilized), 200 µL 1M $MgSO_4$ (filter-sterilized), 20 mL 5x M9 salts (autoclaved), and 1 mL 1 M glucose (filter-sterilized). Antibiotic(s), sodium citrate, NIC, and/or G6P (all filter-sterilized) were added to autoclaved media once the bottles had cooled enough to handle while still being warm to touch. Filter-sterilized antibiotic stock solutions were prepared at the following concentrations: 5 mg/mL CIP, 10 mg/mL FOS, 5 mg/mL TOB, 10 mg/mL DCS, 100 mg/mL ampicillin (AMP), 50 mg/mL kanamycin (KAN). Filter-sterilized sodium citrate was prepared as a 1 M stock solution, filter-sterilized G6P was prepared at 25 mg/mL stock solution, and filter-sterilized NIC was prepared as a 20 mg/mL stock solution.

Electrocompetent cells

To make cells electrocompetent for transformation with plasmids, 2 mL of cells grown to mid-log phase (OD_{600} ~0.4-0.6) were added to sterile microcentrifuge tubes and chilled on ice for 10-15 minutes. All subsequent centrifugation steps were at 5,000 rpm for 5 minutes at $4^{o}C$, and sterile 1% and 10% glycerol solutions were prepared by diluting filter-sterilized 50% glycerol in autoclaved water. The chilled cultures were centrifuged, the supernatant was removed, and the pellets were washed with 1 mL cold 1% glycerol (wash step 1). The cultures were then centrifuged again, the supernatant was removed, and the pellets were washed with 1 mL cold 10% glycerol (wash step 2). The second wash step was then repeated. Finally, the cultures were centrifuged, the supernatant was removed, and cells pellets were resuspended in 100 µL cold 10% glycerol. Those

concentrated cultures were then mixed with appropriate plasmid or linear DNA and kept on ice for at least 30 minutes prior to electroporation.

P1 bacteriophage transduction

For P1 phage generation, the appropriate donor strain from the Keio collection (1) was grown from a -80°C, 25% glycerol stock overnight in 2 mL LB at 37°C. Overnight donor cultures were diluted 10^2-fold in LB supplemented with 7 mM $CaCl_2$ (from filter-sterilized 1M $CaCl_2$ stock solution) and 12 mM $MgSO_4$ (from filter-sterilized 1M $MgSO_4$ stock solution) in sterile 15 mL polypropylene centrifuge tubes. Fifty µL of WT MG1655 P1 phage stock were added, and tubes were incubated at 37°C for 20 minutes without shaking, and then for 2 hours with shaking. An additional 150 µL of overnight donor culture was then added, and the tube was again incubated at 37°C for 20 minutes without shaking, followed by 37°C incubation for 2 hours with shaking. Following the second round of incubation, 1 mL of chloroform were added, and cultures were centrifuged for 15 minutes at 3,000 rpm. The phage-containing supernatants were then stored at 4°C in sterile microcentrifuge tubes as the phage stock for subsequent P1 phage transductions.

To conduct P1 transductions, 400 µL of phage stocks were added to 1 mL of LB supplemented with 7 mM $CaCl_2$ and 12 mM $MgSO_4$ (prepared as described above) in sterile microcentrifuge tubes to generate concentrated phage cultures. The concentrated phage cultures were mixed by pipetting, and then 100 µL were added to additional 1mL aliquots of LB supplemented with 7 mM $CaCl_2$ and 12 mM MgSO4 in sterile microcentrifuge tubes to generate dilute cultures. One hundred µL of appropriate recipient strains (grown from a -80°C 25% glycerol stock overnight in 2 mL LB at 37°C) were then added to both the concentrated phage and dilute phage tubes, which were then incubated at 37°C with shaking for 1 hour. The tubes were then centrifuged at 15,000 rpm for 3 minutes, supernatant were removed until ~100 µL remained in the

tubes, and pellets of both the concentrated and dilute phage tubes were resuspended and combined in the remaining supernatant. The tubes were then centrifuged again, supernatant was removed until ~100 µL remained, and the pellet was resuspended in the remaining supernatant and spread onto LB agar plates supplemented with 50 µg/mL KAN and 25 mM sodium citrate. Plates were incubated at 37 °C for 16 hours, after which individual colonies were colony purified on LB agar containing 50 µg/mL KAN. Colonies were then picked, stocked in filter-sterilized 50% glycerol (final concentration of 25% glycerol), and verified for presence of kanamycin resistance cassette (*kanR*) at the correct gene locus by PCR using primers listed in Table S4.

Curing *kanR* cassettes from deletion strains

All genetic deletion strains generated by P1 transduction were cured of *kanR* by FLP-recombination via transformation with pCP20 (1). Strains were grown from a -80°C, 25% glycerol stock overnight in 2 mL LB at 37°C with shaking. Overnight cultures were then diluted 10^2-fold in 2 mL SOB and grown to mid-log phase (OD_{600} ~0.4-0.6) at 37°C with shaking. Cells were then made electrocompetent as described above before mixing with ~20-100 ng of pCP20. Cultures were then transferred to sterile 0.1 cm electroporation cuvettes and electroporated at 1.8 kV. Electroporated cultures were then immediately recovered in 1 mL SOC for 1 hour at 30°C with shaking before plating on LB agar supplemented with 100 µg/mL AMP. Plates were incubated overnight at 30°C for up to 24 hours. Individual colonies were then purified on antibiotic-free LB agar and incubated at 42°C overnight. Individual colonies were picked, stocked in filter-sterilized 50% glycerol (final concentration of 25% glycerol), and confirmed for the absence of *kanR* and presence of the FRT scar sequence by colony PCR (Table S3) and lack of growth in 50 µg/mL KAN at 37°C. Cultures were also screened for inability to grow in 100 µg/mL AMP at 30°C to confirm loss of pCP20.

Tn7 transposition

Insertions at the Tn7 transposase attachment site were conducted according to the protocol published by McKenzie and Craig (2). Wild-type MG1655 or UTI89 were grown from a -80°C, 25% glycerol stock in 2 mL LB at 37°C for 16 hours. Overnight cultures were diluted 10^2-fold in 2 mL SOB and grown to mid-log phase (OD_{600} ~0.4-0.6) at 37°C with shaking. Cells were then made electrocompetent as described above before mixing with ~50-100 ng of pGRG25 or appropriate derivative (Table S3). Cultures were then transferred to sterile 0.1 cm electroporation cuvettes and electroporated at 1.8 kV. Electroporated cultures were then immediately recovered in 1 mL SOC for 1 hour at 30-32°C while shaking before plating on LB agar supplemented with 100 µg/mL AMP. Plates were incubated overnight at 30-32°C, and individual colonies were purified on LB supplemented with 100 µg/mL AMP and incubated again at 30-32°C. Individual colonies were then inoculated into 2 mL LB and incubated at 30-32 °C with shaking. After 3 hours, cultures were then supplemented with filter-sterilized 1 M L-arabinose to a final concentration of 0.1% and continued to incubate at 30-32°C with shaking overnight. Overnight cultures were then diluted ~10^6-fold, 100 µL were spread on LB agar, and plates were incubated at 42°C overnight to allow for plasmid loss. Individual colonies were picked, stocked in filter-sterilized 50% glycerol (final concentration of 25% glycerol), and confirmed for presence of desired insert at *attTn7* by PCR (Table S3). Stocks were also confirmed for loss of plasmid by lack of growth in 100 µg/mL AMP at 30-32°C.

CRISPR Optimized MAGE Recombineering (CRMAGE)

CRISPR/Cas9 and λ-Red recombineering (CRMAGE) was conducted according to protocol by Ronda and colleagues (3) to introduce a premature STOP and/or PAM mutation(s) in *ddlB* of UTI89. Because expression of the plasmid-encoded CRISPR machinery is regulated by

the *tet* operator, constitutive expression of *tetR* by the parent strain was required. Thus, UTI89 *attTn7*::P_{N25}-*tetR* was first generated using pGRG25 P_{N25}-*tetR* (Table S2) according to Tn7 transposition protocol described above. UTI89 *attTn7*::P_{N25}-*tetR* was then transformed with pMA7CR-2.0. Overnight cultures were diluted 10^2-fold in 2 mL SOB and grown to mid-log phase (OD_{600} ~0.4-0.6) at 37°C while shaking. Cells were then made electrocompetent as described above before mixing with ~100 ng pMA7CR-2.0. Cultures were then transferred to sterile 0.1 cm electroporation cuvettes and electroporated at 1.8 kV. Electroporated cultures were then immediately recovered in 1 mL SOC for 1 hour at 37°C while shaking before plating on LB agar supplemented with 100 µg/mL AMP. Plates were incubated at 37 °C for 16 hours, and individual colonies then glycerol stocked in LB media supplemented with 100 µg/mL AMP.

To introduce the STOP and/or PAM mutation(s), UTI89 *attTn7*::P_{N25}-*tetR* pMA7CR-2.0 was grown from a -80°C 25% glycerol stock in 2 mL LB supplemented with 100 µg/mL AMP for 16 hours at 37 °C. The overnight culture was then diluted 10^2-fold in 15 mL LB supplemented with 100 µg/mL AMP in a baffled flask and grown to an OD_{600} ~0.5 at 37°C with shaking. Filter-sterilized 1 M L-arabinose was then added to a final concentration of 0.2%, and the flask was incubated for an additional 15 minutes at 37°C with shaking. The flask was then placed on ice for 20 minutes before the entire culture was transferred to a 50 mL conical centrifuge tube. All subsequent centrifugation steps were at 4,000 rpm for 10 minutes at 4°C. The culture was centrifuged, the entire supernatant was removed, and the cell pellet was resuspended in 35 mL cold water (previously sterilized by autoclaving). The culture was centrifuged again, the entire supernatant was removed, and the cell pellet was resuspended in 15 mL cold water. The culture was centrifuged again, the entire supernatant was removed, and the cell pellet was resuspended in 1 mL cold water. The culture was centrifuged again, the entire supernatant was removed, and the

cell pellet was resuspended in 800 µL cold water. Fifty µL of that cell culture was then aliquoted into sterile microcentrifuge tubes, and 0.5 µL of appropriate 10 µM mutagenic oligo (prepared in autoclaved water) along with ~250 ng of pMAZSK $gRNA_{ddlB}$ was added to each aliquot. The cultures were then chilled on ice for ~30 minutes before being transferred to sterile 0.1 cm electroporation cuvettes and electroporated at 1.8 kV. Electroporated cultures were then immediately recovered in 1 mL LB supplemented with 100 µg/mL AMP at 37°C while shaking. After 1 hour of recovery, KAN was added to each culture to a final concentration of 50 µg/mL to retain pMAZSK $gRNA_{ddlB}$, and the cultures were incubated for an additional 2 hours at 37°C while shaking. After 3 hours total incubation, aTET was added to each culture to a final concentration of 200 ng/mL to induce CRISPR/Cas9 and gRNA machinery, and cultures were incubated for an additional 2 hours at 37°C while shaking. Fifty µL of each culture were then removed, 5-fold dilutions were spotted on LB agar supplemented with 100 µg/mL AMP, 50 µg/mL KAN, and 200 ng/mL aTET, and plates were incubated at 37 °C for 16 hours to select for transformants. Individual colonies were purified non-selectively on LB agar plates and incubated again at 37 °C for 16 hours. Colonies were then stocked in filter-sterilized 50% glycerol (final concentration of 25% glycerol) and confirmed for presence of desired mutation(s) by PCR and Sanger Sequencing. To cure of pMA7CR-2.0 and pMAZSK $gRNA_{ddlB}$, stocks confirmed to have desired mutation(s) were purified non-selectively on LB agar and screened for loss of ability to form colonies on LB media supplemented with 100 µg/mL AMP or 50 µg/mL KAN. Plasmid cure was also confirmed by absence of PCR amplicon using primers which anneal to each plasmid (Table S4).

Broth Microdilution

Broth microdilution was performed to determine the DCS minimum inhibitory concentrations (MIC) for MG1655 and UTI89 (4). For all steps below, “minimal media” refers to

M9 media supplemented with 10 mM glucose (for MG1655) or M9 media supplemented with 10 mM glucose and 1 µg/mL NIC (for UTI89). Cultures of MG1655 or UTI89 were grown overnight in 2 mL LB for 16 hours at 37°C. After overnight growth, 500 µL of cultures were pelleted at 15,000 RPM for 3 minutes, the supernatant was removed, and pellets were resuspended in 500 µL minimal media. Samples were then diluted to an OD_{600} of ~0.0001 (~10^5 CFU/mL), and 75 µL of those diluted cultures were added to 75 µL of minimal media containing 2-fold dilutions of DCS in wells of a flat-bottom 96-well plate. Additionally, 75 µL of diluted cultures (OD_{600} ~0.0001) and 75 µL of cell-free minimal media were each added to wells containing 75 µL of DCS-free minimal media to serve as untreated growth controls and sterility controls, respectively. The plates were then covered with sterile Breathe-Easy membrane adhesive strips and incubated at 37°C while shaking for 24 hours. After overnight incubation, cultures in all wells were diluted in fresh minimal media, and the OD_{600} were measured. The minimum inhibitory concentrations (MIC) were determined to be the lowest concentrations at which OD_{600} were significantly different from that in the untreated growth control and not significantly different from all OD_{600} measured at higher DCS concentrations.

<u>Derivation of RT-qPCR transcript quantification formula</u>

To arrive at our expression for quantifying *cycA* and *ddlB* transcript levels in our 0-, 1-, and 2-copy mutants, we first considered that the measured C_T value for each qPCR well indicates the amplification cycle at which the measured fluorescence of that reaction reached the same threshold value (where the threshold fluorescence value is set within the geometric amplification portion of all amplification curves) (5). Thus, we can relate the copy number abundances of any two genes (*e.g.*, for gene "A" and gene "B") between two qPCR wells by the general equation:

$$K_A n_A E_A^{C_T^A} = K_B n_B E_B^{C_T^B} \qquad (1)$$

n_A *and* n_B ≡ No. of *DNA copies of genes* A and B, respectively, initially present in the qPCR well

E_A *and* E_B ≡ Efficiency of amplification by primers which anneal to transcripts A and B, respectively

C_T^A *and* C_T^B ≡Measured cycle at which the reactions amplifying genes A and B, respectively, within a given sample have reached the threshold fluorescence value

K_A*and* K_B ≡ Proportionality constants for converting DNA copy number of genes A and B, respectively, to a fluorescence value

Since SYBR Green dye binds in a sequence-independent manner to the minor grooves of dsDNA (5), and the PCR products used here were of comparable sizes (135 bp-175 bp), we assume that $K_{cycA} = K_{ddlB} = K_{phzM}$.

Applying the above assumption and equation (1) to our RT-qPCR experiments, we can relate the amplification of the cDNA for our GOI to that of the *phzM* external control for each RT^+ sample by:

$$(n_{gene} + \gamma)E_{gene}^{C_T^{gene,RT^+}} = (n_{phzM} + \rho)E_{phzM}^{C_T^{phzM,RT^+}} \qquad (2)$$

n_{gene} ≡ No. transcripts of GOI initially present in reaction (present in RT^+ sample only)

n_{phzM} ≡ No. *t*ranscripts of *phzM* initially present in reaction (present in RT+ sample only)

γ ≡ No. residual gDNA copies potentially present in reaction (for RT^+ or RT^- samples)

ρ ≡ No. residual pET11a PT7-*phzM* copies potentially present in reaction (for RT^+ or RT^- samples)

C_T^{gene,RT^+} ≡ threshold cycle for reaction containing RT^+ sample and primers annealing to GOI

C_T^{phzM,RT^+} ≡ threshold cycle for reaction containing RT^+ sample and primers annealing to p*hzM*

E_{gene} ≡ measured efficiency of primers annealing to the GOI

E_{phzM} ≡ measured efficiency of primers annealing to *phzM*

Similarly, we can relate the measurements of the qPCR wells containing each RT^+ sample to their corresponding RT^- sample for each primer pair by:

$$\gamma E_{gene}^{C_T^{gene,RT^-}} = (n_{gene} + \gamma) E_{gene}^{C_T^{gene,RT^+}} \quad (3)$$

$$\rho E_{phzM}^{C_T^{phzM,RT^-}} = (n_{phzM} + \rho) E_{phzM}^{C_T^{phzM,RT^+}} \quad (4)$$

C_T^{gene,RT^-} ≡ threshold cycle for reaction containing RT$^-$ sample and primers annealing to GOI

C_T^{phzM,RT^-} ≡ threshold cycle for reaction containing RT$^-$ sample and primers annealing to p*hzM*

Now, we have 3 independent equations [(2), (3), and (4)] with 4 unknowns [$n_{gene}, n_{phzM,}\ \rho, \gamma$] from which we can isolate an expression for $\left(\frac{n_{gene}}{n_{phzM}}\right)_{sample}$.

Rearranging (3) to isolate γ:

$$\gamma E_{gene}^{C_T^{gene,RT^-}} = (n_{gene} + \gamma) E_{gene}^{C_T^{gene,RT^+}} \quad (3)$$

$$\gamma = (n_{gene} + \gamma) E_{gene}^{\left\{C_T^{gene,RT^+} - C_T^{gene,RT^-}\right\}} \quad (3a)$$

$$\gamma(1 - E_{gene}^{\left\{C_T^{gene,RT^+} - C_T^{gene,RT^-}\right\}}) = n_{gene}(E_{gene}^{\left\{C_T^{gene,RT^+} - C_T^{gene,RT^-}\right\}}) \quad (3b)$$

$$\gamma = n_{gene} \left[\frac{E_{gene}^{\left\{C_T^{gene,RT^+} - C_T^{gene,RT^-}\right\}}}{1 - E_{gene}^{\left\{C_T^{gene,RT^+} - C_T^{gene,RT^-}\right\}}} \right] \quad (3c)$$

Rearranging (4) to isolate ρ:

$$\rho E_{phzM}^{C_T^{phzM,RT^-}} = (n_{phzM} + \rho) E_{phzM}^{C_T^{phzM,RT^+}} = \quad (4)$$

$$\rho = (n_{phzM} + \rho) E_{phzM}^{\left\{C_T^{phzM,RT^+} - C_T^{phzM,RT^-}\right\}} \quad (4a)$$

$$\rho(1 - E_{phzM}^{\left\{C_T^{phzM,RT^+} - C_T^{phzM,RT^-}\right\}}) = n_{phzM}(E_{phzM}^{\left\{C_T^{phzM,RT^+} - C_T^{phzM,RT^-}\right\}}) \quad (4b)$$

$$\rho = n_{phzM}\left[\frac{E_{phzM}^{\left\{C_T^{phzM,RT^+} - C_T^{phzM,RT^-}\right\}}}{1 - E_{phzM}^{\left\{C_T^{phzM,RT^+} - C_T^{phzM,RT^-}\right\}}}\right] \quad (4c)$$

Substituting (3c) and (4c) into equation (2):

$$\left(n_{gene} + n_{gene}\left[\frac{E_{gene}^{\left\{C_T^{gene,RT^+} - C_T^{gene,RT^-}\right\}}}{1 - E_{gene}^{\left\{C_T^{gene,RT^+} - C_T^{gene,RT^-}\right\}}}\right]\right)E_{gene}^{C_T^{gene,RT^+}} = \left(n_{phzM} + n_{phzM}\left[\frac{E_{phzM}^{\left\{C_T^{phzM,RT^+} - C_T^{phzM,RT^-}\right\}}}{1 - E_{phzM}^{\left\{C_T^{phzM,RT^+} - C_T^{phzM,RT^-}\right\}}}\right]\right)E_{phzM}^{C_T^{phzM,RT^+}} \quad (5)$$

Grouping coefficients of n_{gene} and n_{phzM} and simplifying:

$$n_{gene}\left(1 + \left[\frac{E_{gene}^{\left\{C_T^{gene,RT^+} - C_T^{gene,RT^-}\right\}}}{1 - E_{gene}^{\left\{C_T^{gene,RT^+} - C_T^{gene,RT^-}\right\}}}\right]\right)E_{gene}^{C_T^{gene,RT^+}} = n_{phzM}\left(1 + \left[\frac{E_{phzM}^{\left\{C_T^{phzM,RT^+} - C_T^{phzM,RT^-}\right\}}}{1 - E_{phzM}^{\left\{C_T^{phzM,RT^+} - C_T^{phzM,RT^-}\right\}}}\right]\right)E_{phzM}^{C_T^{phzM,RT^+}} \quad (5a)$$

$$n_{gene}\left[\frac{1}{1 - E_{gene}^{\left\{C_T^{gene,RT^+} - C_T^{gene,RT^-}\right\}}}\right]E_{gene}^{C_T^{gene,RT^+}} = n_{phzM}\left[\frac{1}{1 - E_{phzM}^{\left\{C_T^{phzM,RT^+} - C_T^{phzM,RT^-}\right\}}}\right]E_{phzM}^{C_T^{phzM,RT^+}} \quad (5b)$$

$$\left(\frac{n_{gene}}{n_{phzM}}\right)_{sample} = \left[\frac{E_{phzM}^{C_T^{phzM,RT^+}}}{E_{gene}^{C_T^{gene,RT^+}}}\right]\left[\frac{1 - E_{gene}^{\left\{C_T^{gene,RT^+} - C_T^{gene,RT^-}\right\}}}{1 - E_{phzM}^{\left\{C_T^{phzM,RT^+} - C_T^{phzM,RT^-}\right\}}}\right] \quad (5c)$$

Finally, because the same amount of *phzM* was added to each sample before RNA extraction for every replicate, we can assume that n_{phzM} is constant across all samples and independent replicates. However, since $n_{gene} \propto no.\,cells\,harvested \propto OD_{600}^{sample}$, an OD_{600}-based normalization factor must be applied to (5c) to allow for comparison of n_{gene} across independent harvest replicates:

$$\left(\frac{n_{gene}}{n_{phzM}}\right)_{sample} = \left[\frac{E_{phzM}^{C_T^{phzM,RT^+}}}{E_{gene}^{C_T^{gene,RT^+}}}\right]\left[\frac{1-E_{gene}^{\left\{C_T^{gene,RT^+}-C_T^{gene,RT^-}\right\}}}{1-E_{phzM}^{\left\{C_T^{phzM,RT^+}-C_T^{phzM,RT^-}\right\}}}\right]\left[\frac{OD_{600}^{min}}{OD_{600}^{sample}}\right] \quad (6)$$

where OD_{600}^{sample} is the final OD_{600} to which the sample had been adjusted after addition to RNAProtect during harvest, and OD_{600}^{min} is the minimum value of the set of OD_{600}^{sample} for all 3 harvest replicates ($OD_{600}^{min} = 0.1$ for both *cycA* and *ddlB* strain sets). Thus, we can use equation (6) to compare GOI transcript levels among different samples and between replicates because n_{phzM} is constant across all samples and replicates and n_{gene} is normalized to the same OD_{600}^{min} such that the only variations in n_{gene} would be a result of expression differences of the GOI in each sample.

References

1. Baba T, Ara T, Hasegawa M, Takai Y, Okumura Y, Baba M, Datsenko KA, Tomita M, Wanner BL, Mori H. 2006. Construction of *Escherichia coli* K-12 in-frame, single-gene knockout mutants: the Keio collection. *Molecular Systems Biology* 2:2006.0008.
2. McKenzie GJ, Craig NL. 2006. Fast, easy and efficient: site-specific insertion of transgenes into Enterobacterial chromosomes using Tn7 without need for selection of the insertion event. *BMC Microbiology* 6:39.
3. Ronda C, Pedersen LE, Sommer MOA, Nielsen AT. 2016. CRMAGE: CRISPR Optimized MAGE Recombineering. *Scientific Reports* 6:19452.
4. Andrews JM. 2001. Determination of minimum inhibitory concentrations. *Journal of Antimicrobial Chemotherapy* 48(1): 5-16.
5. Dymond JS. 2023. Explanatory Chapter: Quantitative PCR. *Methods in Enzymology* 529:279-89.

Supplemental figures

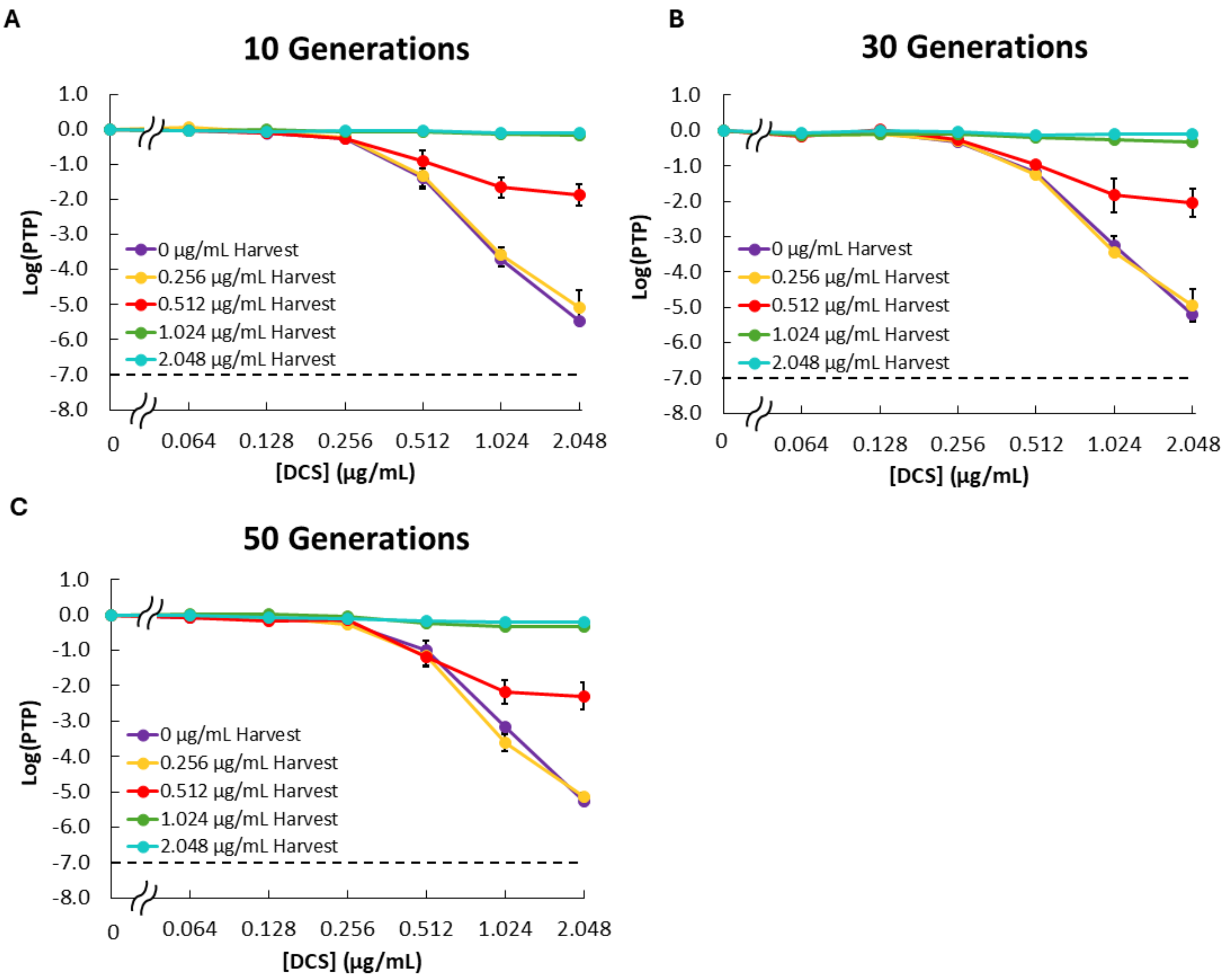

Figure S1. MG1655 DCS minority subpopulation stability through 50 generations of propagation. (A)-(C) Subpopulation stability PAP curves after (A) 10 generations, (B) 30 generations, or (C) 50 generations of propagation without antibiotic. Data represents averages of at least three biological replicates, and error bars represent SEM. Dashed line indicates threshold for heteroresistance.

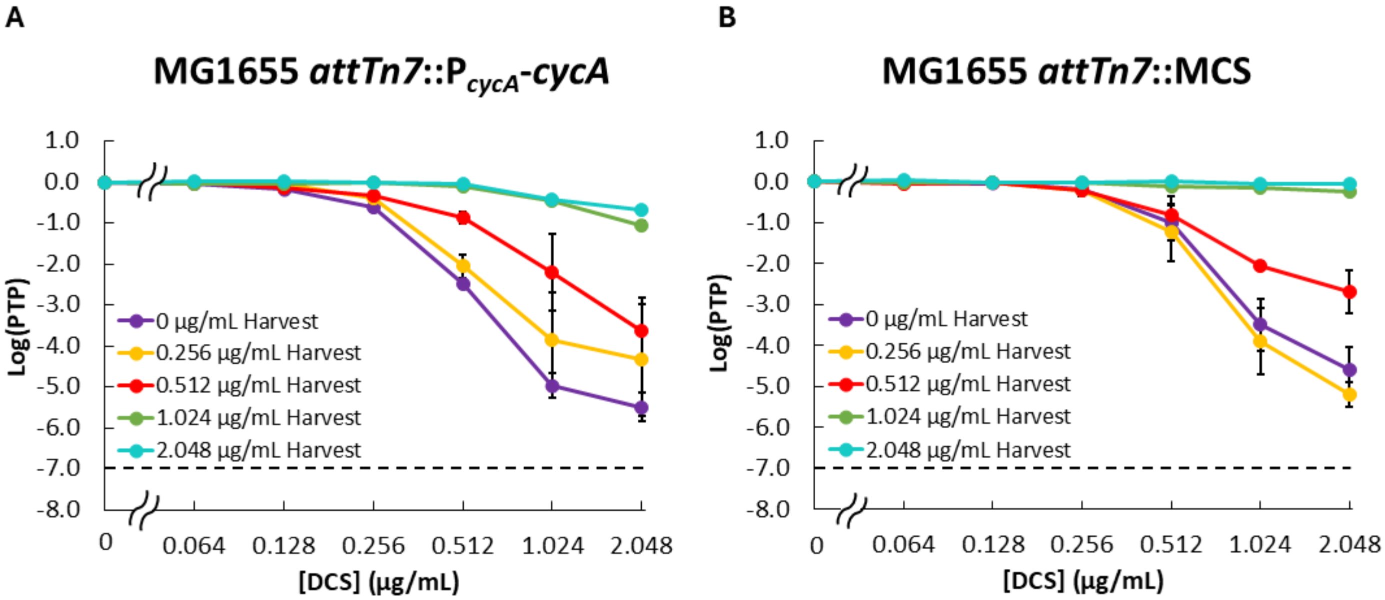

Figure S2. PAP of harvested minority subpopulations of DCS transporter two-copy mutant and control. (A) MG1655 *attTn7*::P_{cycA}-*cycA* and (B) MG1655 *attTn7*::MCS after propagation for 10 generations without antibiotic. Data represents averages of at least three biological replicates, and error bars represent SEM. Dashed line indicates threshold for heteroresistance.

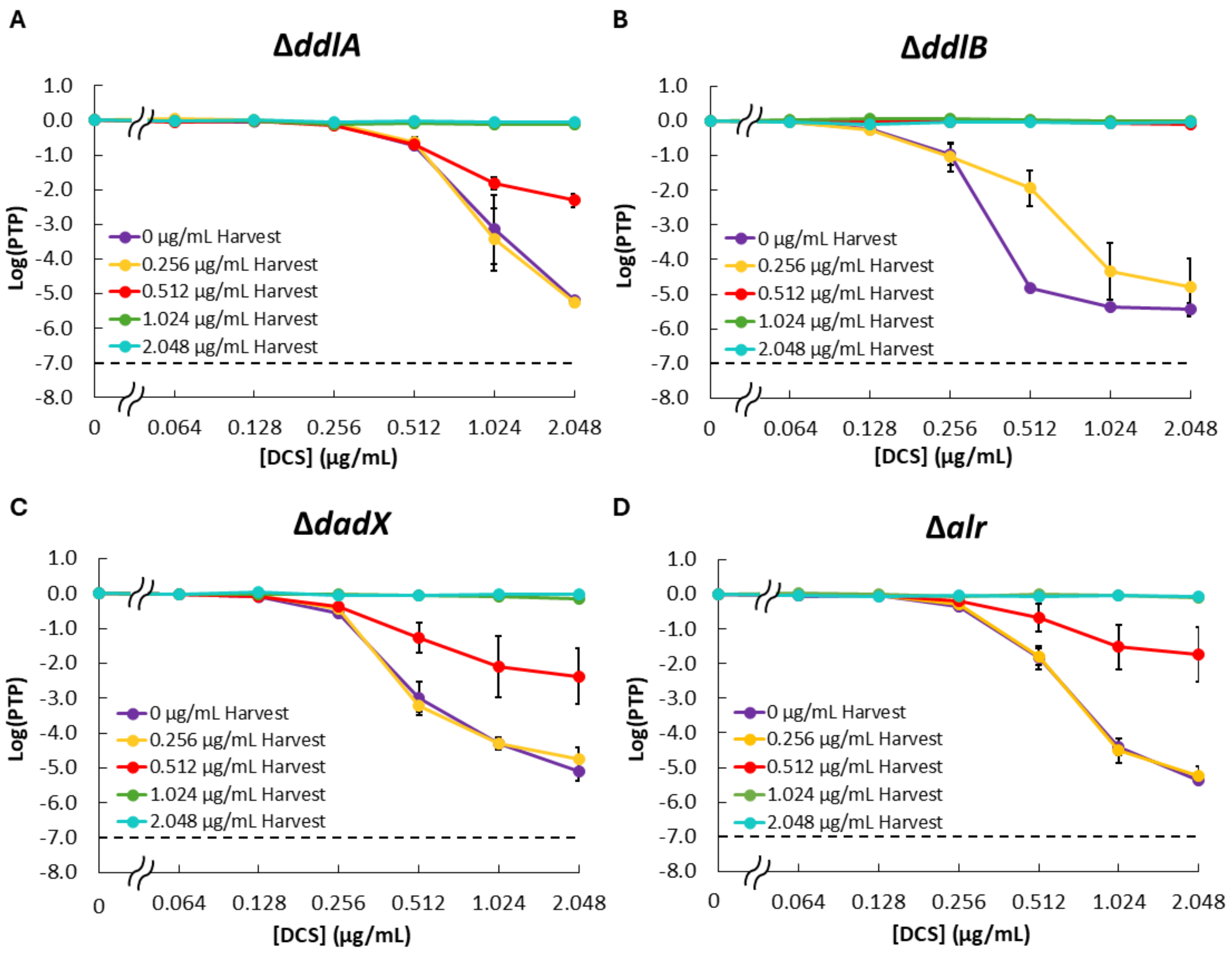

Figure S3. PAP of harvested minority subpopulations of DCS target single-deletion mutants. (A) Δ*ddlA*, (B) Δ*ddlB*, (C) Δ*dadX*, and (D) Δ*alr* of MG1655 after propagation for 10 generations without antibiotic. Data represents averages of at least three biological replicates, and error bars represent SEM. Dashed line indicates threshold for heteroresistance.

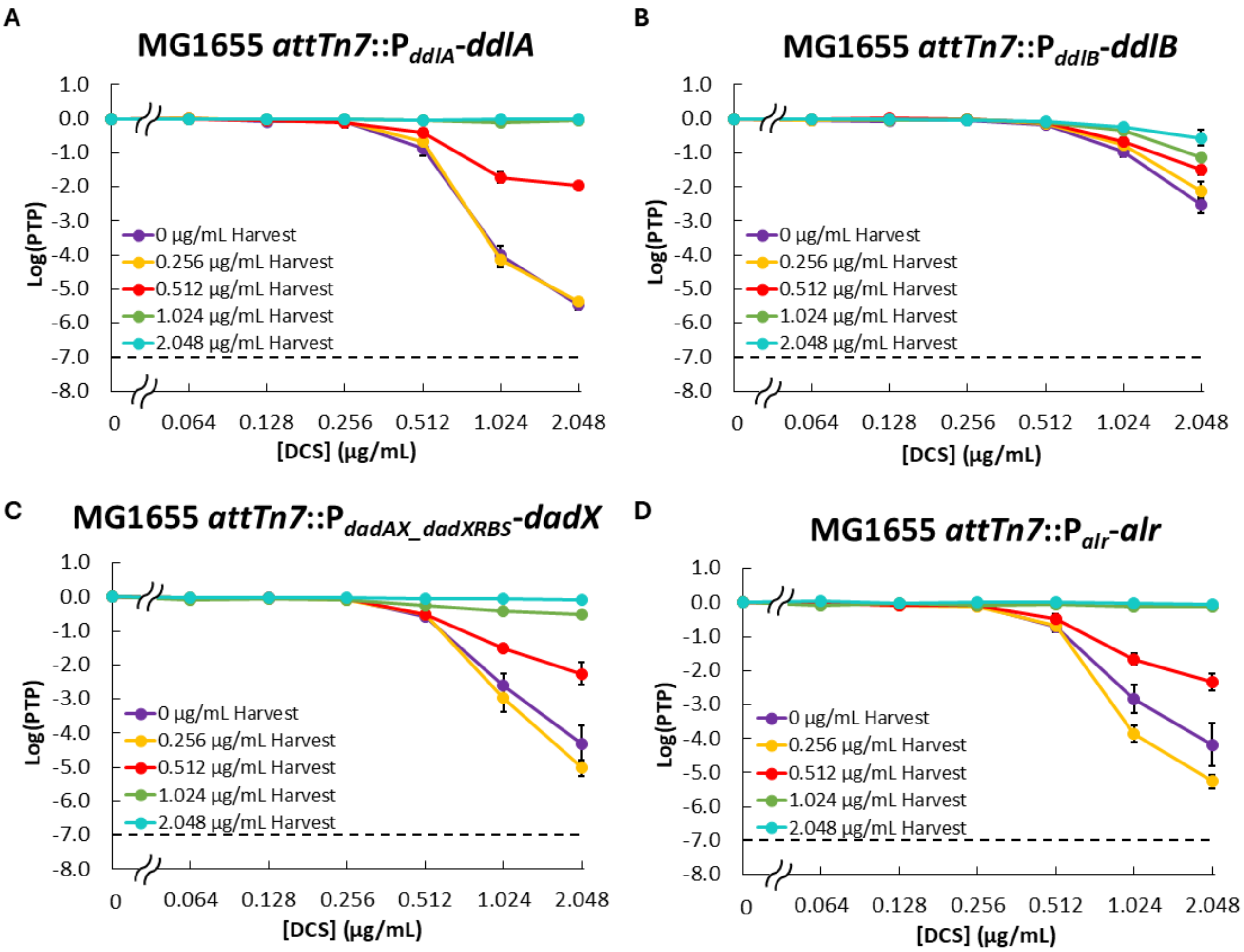

Figure S4. PAP of harvested minority subpopulations of DCS target two-copy mutants. (A) MG1655 *attTn7*::P$_{ddlA}$-*ddlA*, (B) MG1655 *attTn7*::P$_{ddlB}$-*ddlB*, (C) MG1655 *attTn7*::P$_{dadAX_dadXRBS}$-*dadX*, and (D) MG1655 *attTn7*::P$_{alr}$-*alr* after propagation for 10 generations without antibiotic. Note that the two-copy control, MG1655 *attTn7*::MCS, appears in Figure S2. Data represents averages of at least three biological replicates, and error bars represent SEM. Dashed line indicates threshold for heteroresistance.

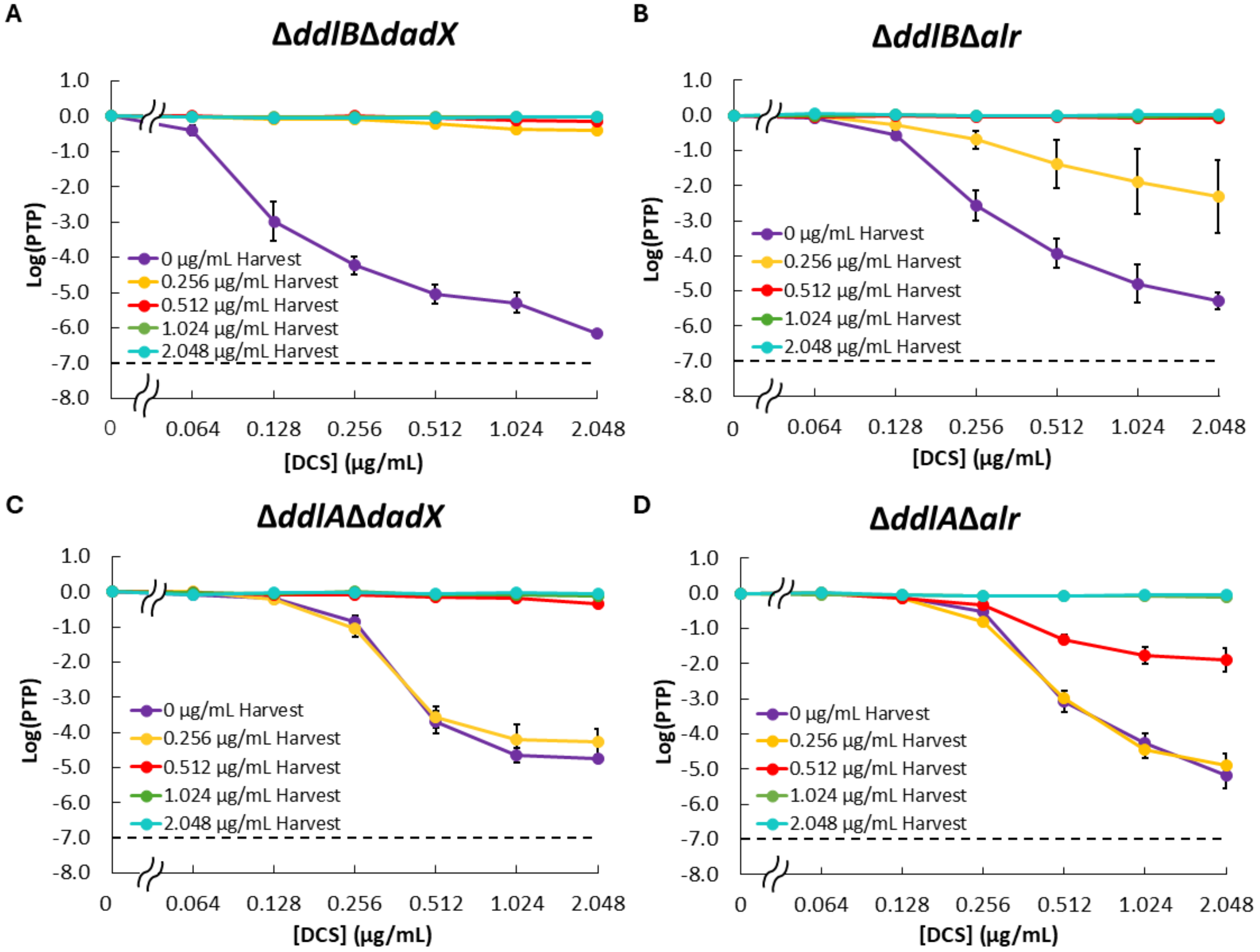

Figure S5. PAP of harvested minority subpopulations of MG1655 DCS target double-deletion mutants. (A) Δ*ddlB*Δ*dadX*, (B) Δ*ddlB*Δ*alr*, (C) Δ*ddlA*Δ*dadX*, and (D) Δ*ddlA*Δ*alr* after propagation for 10 generations without antibiotic. Data represents averages of at least three biological replicates, and error bars represent SEM. Dashed line indicates threshold for heteroresistance.

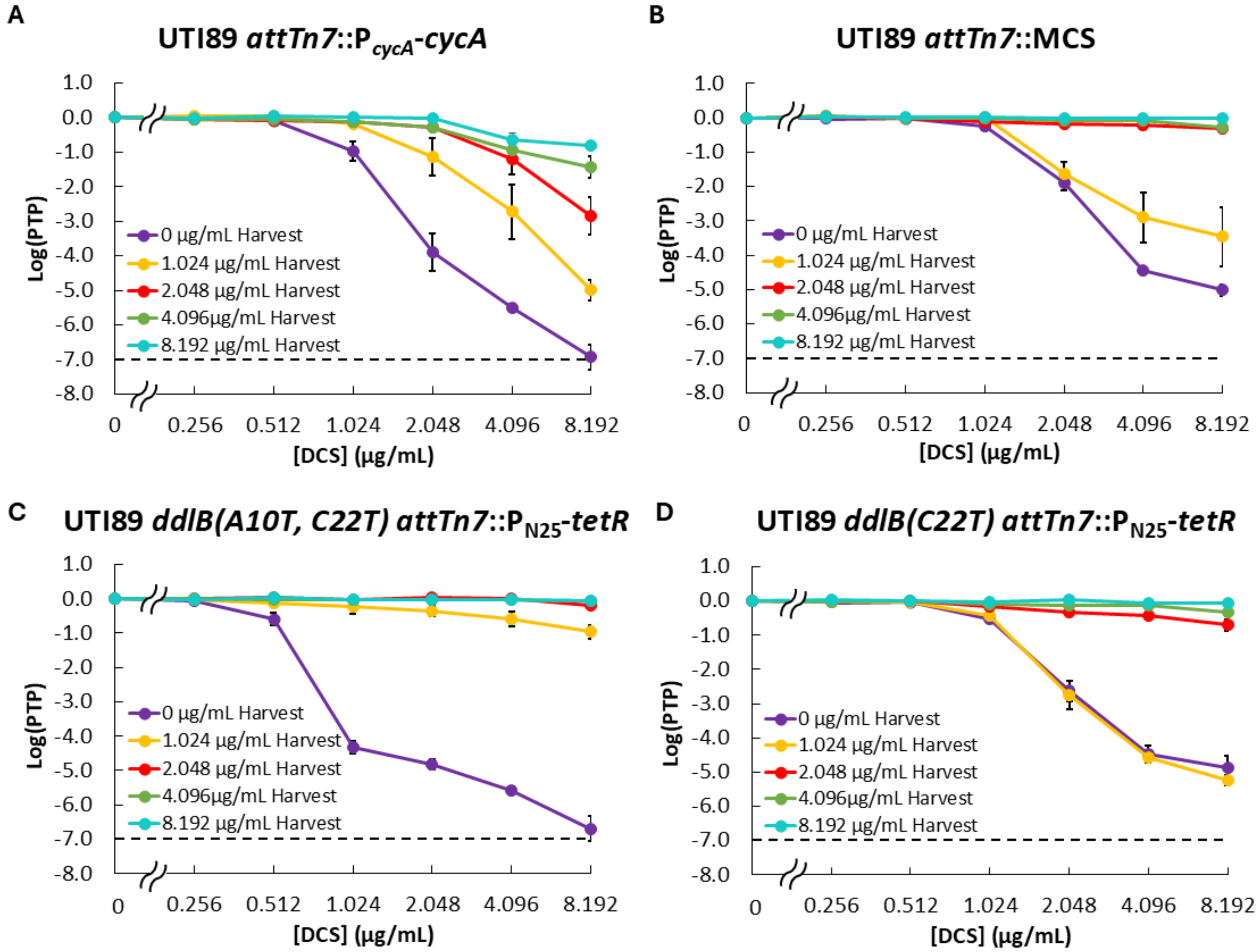

Figure S6. PAP of harvested DCS minority subpopulations of UTI89 mutants. (A) UTI89 *attTn7*::P$_{cycA}$-*cycA*, (B) UTI89 *attTn7*::MCS, (C) UTI89 *ddlB(A10T*, *C22T) attTn7*::P$_{N25}$-*tetR*, and (D) UTI89 *ddlB(C22T) attTn7*::P$_{N25}$-*tetR* after propagation for 10 generations without antibiotic. Data represents averages of at least three biological replicates, and error bars represent SEM. Dashed line indicates threshold for heteroresistance.

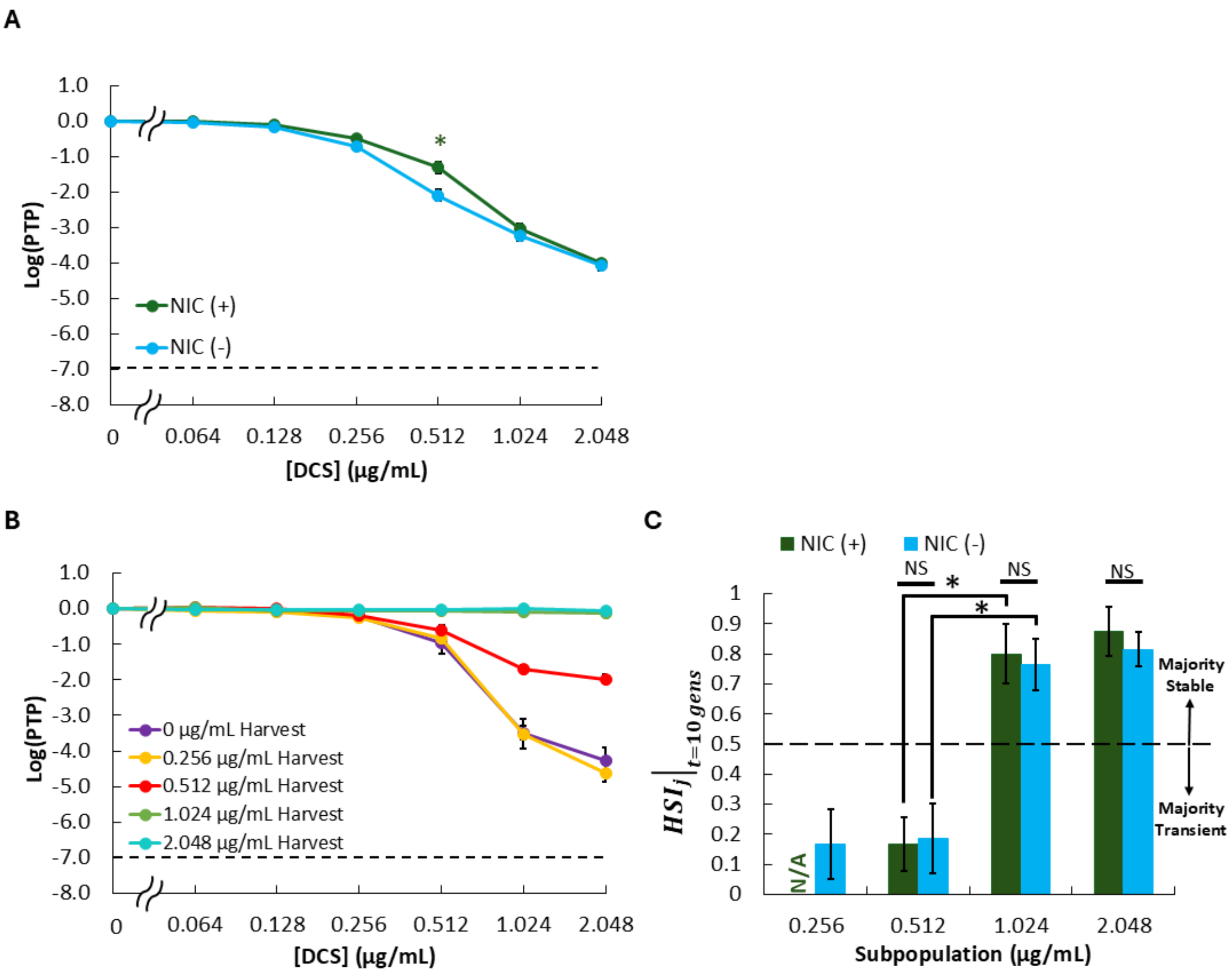

Figure S7. Impact of NIC on DCS heteroresistance and subpopulation stability for MG1655. (A) PAP of MG1655 with (NIC (+)) and without (NIC (-)) the addition of 1 µg/mL NIC in agar plates. (B) Subpopulation stability PAP of MG1655 with NIC added to plates. (C) $HSI_j\big|_{t=10\ gens}$ of MG1655 with (NIC (+)) and without (NIC (-)) the addition of 1 µg/mL NIC in agar plates. Data represents averages of at least three biological replicates, and error bars represent SEM. Dashed lines in (A) and (B) indicate threshold for heteroresistance. Dashed line in (C) delineates the point of transition between subpopulation stability states (majority transient for $HSI_j\big|_t < 0.5$, vs. majority stable for $HSI_j\big|_t > 0.5$). N/A indicates concentration where subpopulation stability was not calculated due to functional limitations of HSI (0xHNIC control subpopulations did not

produce minority subpopulations after propagation during subpopulation stability PAP, $PTP_j^0\big|_t >$ 0.5, for the majority of replicates). Star (*) represents statistical significance by one-way ANOVA and Tukey post hoc analysis ($p < 0.05$). NS = No significance.

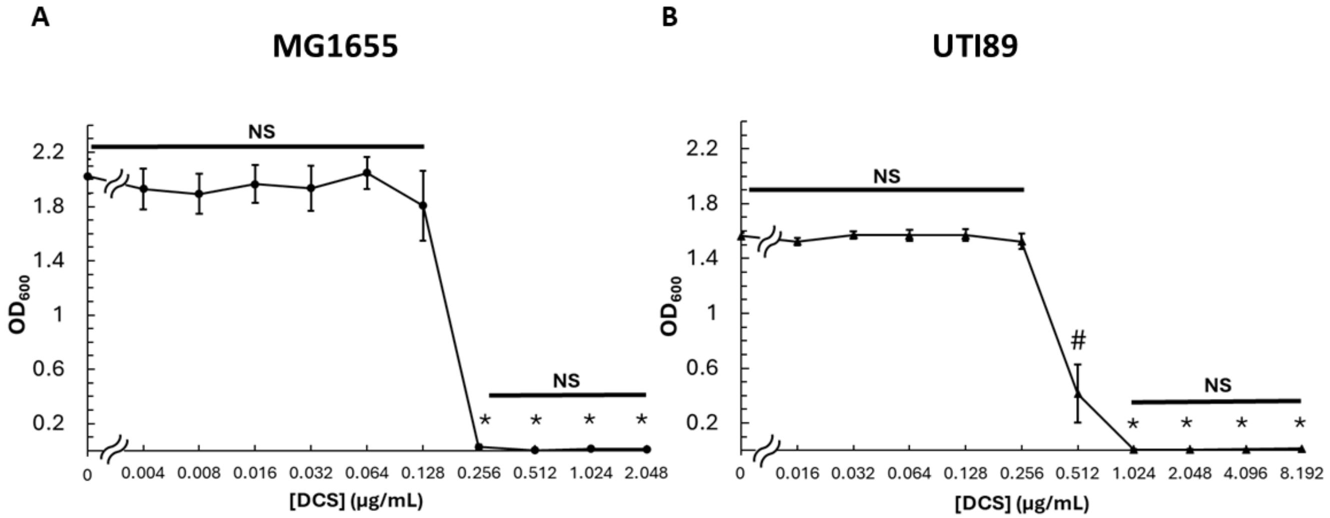

Figure S8. Broth microdulution to determine the DCS MIC for MG1655 and UTI89. Measured OD_{600} of (A) MG1655 or (B) UTI89 after 24 hour incubation in minimumal media containing 2-fold dilutions of DCS. DCS minimum inhibitory concentration (MIC) was determined to be the lowest concentration at which the OD_{600} was significantly different from that measured in DCS-free well and not significantly different from all OD_{600} measured at higher DCS concentrations. Star (*) indicates signficant difference from 0 μg/mL DCS condition by one-way ANOVA with Tukey post hoc analysis ($p < 0.05$). Pound (#) indicates signficant difference from all other DCS concentrations by one-way ANOVA with Tukey post hoc analysis ($p < 0.05$).

Table S1. List of materials used in this study.

Product/component	Manufacturer
Difco LB Broth, Miller (Luria-Bertani) (Premix)	Thermo Fisher
BBL Cation-Adjust Mueller Hinton II Agar Premix	Thermo Fisher
Tryptone	Fisher Bioreagents
Yeast Extract	Fisher Bioreagents
Agar (80-100 mesh)	Fisher Bioreagents
D-glucose	Fisher Chemical
L-arabinose	Alfa Aesar
Glycerol	Sigma Aldrich
Chlorform	Fisher Chemical
Sodium Citrate Dihydrate ($Na_3C_6H_5O_7$*$2H_2O$)	Fisher Scientific
Potassium Chloride (KCl)	Fisher
Sodium Chloride (NaCl)	Fisher Bioreagents
Calcium Chloride Dihydrate ($CaCl_2$)	Fisher Bioreagents
Magnesium Sulfate Heptahydrate ($MgSO_4$*$7H_2O$)	MP Biochemicals
Sodium Phosphate Dibasic Heptahydrate (Na_2HPO_4*$7H_2O$)	MP Biochemicals
Potassium dihyrogen Phosphate (KH_2PO_4)	Alfa Aesar
Ammonium Chloride (NH_4Cl)	MP biochemicals
Gluose-6-phosphate (G6P)	Sigma
Nicotinamide (NIC)	Sigma Aldrich
Ciprofloxacin (CIP)	Sigma
Fosfomycin disodium salt (FOS)	Sigma Aldrich
Tobramycin sulfate (TOB)	Sigma Aldrich
D-cycloserine (DCS)	Sigma Aldrich
Anhydrotetracycline hydrochloride (aTET)	Sigma Aldrich
Kanamycin sulfate (KAN)	Gibco
Ampicillin (AMP)	Acros Organics
Lysozyme, chicken egg white	Thermo Fisher
Tris Hydrochloride (1 M, pH 8.0, DNase, RNase, and Protease Free)	Fisher Bioreagents
Ethylenediamine Tetraacetate Acid (EDTA) (for DNA work) (0.5 M, pH 8.0, RNase and Protease Free)	Fisher Bioreagents
Water (nuclease-free, 0.1 µm filtered, for molecular biology)	Sigma Aldrich
Gibson Assembly Master Mix	New Englad Biolabs
Phusion High Fidelity PCR Kit	New Englad Biolabs
Taq DNA Polymerase	New Englad Biolabs
EcoRI-HF Restriction Enzyme	New Englad Biolabs
10x rCutSmart Buffer	New Englad Biolabs
Q5 Site-Directed Mutagenesis Kit	New Englad Biolabs
HiScibe T7 RNA Synthesis Kit	New Englad Biolabs
TaqMan Reverse Transcription Reagents	Thermo Fisher Scientific
Power SYBR Green qPCR Master Mix	Applied Biosystems
Qiaprep Spin Miniprep Kit	Qiagen
Qiaquick Gel Extraction Kit	Qiagen
Dneasy Blood & Tissue Kit	Qiagen
RNase-free DNase Kit	Qiagen
RNeasy Mini Kit	Qiagen
RNAprotect Bacteria Reagent	Qiagen

Table S2. List of plasmids used in this study.

Plasmid	Relevant description†	PCR/Sequence Check(s)*	Source
pUA66	Contains promoterless *gfp* with rrnB T1 terminator, *kanR* resistance cassette, and pSC101 ori.	N/A	[1]
pGRG25	Contains Tn7 Transposition machinery (*tnsABCD*) with a multiple cloning sequence (MCS) between left (Tn7L) and right (Tn7R) transposition arms, *ampR* resistance cassette, and temperature-sensitive Rep101 ori.	N/A	[2]
pCP20	Contains FLP-recombinase machinery, *ampR* resistance cassettes, and temperature sensitive Rep101 ori.	N/A	[3]
pMA7CR-2.0	Contains λ-Red recombinase machinery under the P_{BAD}, CRISPR/Cas9 expression machinery under P_{Ltet}, *ampR* resistance cassette, and ColE1 ori.	N/A	[4]
pMAZSK	Contains gRNA expression machinery under P_{Ltet} promoter, *kanR* resistance casesstte, and ColA ori.	N/A	[4]
pET11a P_{T7}-*phzM*	Contains *phzM* between T7 promoter and terminator (all upstream of an EcoRI resistriction site), *ampR* resistance cassette, and ColE1 ori.	N/A	[5,6]
pUA66 P_{cycA}-*cycA*	Constructed via 2-Fragment Gibson Assembly of P_{cycA}-*cycA* (PCR-amplified from MG1655 gDNA using primer pair 2) and pUA66 backbone (PCR-amplified from pUA66 using primer pair 1).	Verified presence of insert by PCR using primer pairs 20 and 21. Confirmed sequence of insert by Sanger sequencing using each primer of pair 20.	This work
pUA66 P_{ddlA}-*ddlA*	Constructed via 2-Fragment Gibson Assembly of P_{ddlA}-*ddlA* (PCR-amplified from MG1655 gDNA using primer pair 3) and pUA66 backbone (PCR-amplified from pUA66 using primer pair 1).	Verified presence of insert by PCR using primer pairs 20 and 22. Confirmed sequence of insert by Sanger sequencing using each primer of pair 20.	This work
pUA66 P_{ddlB}-*ddlB*	Constructed via 3-Fragment Gibson Assembly of the leading promoter of the *ddlB* operon and *ddlB* ORF (PCR-amplified from MG1655 gDNA using primer pairs 4 and 5, respectively) and the pUA66 backbone (PCR-amplified from pUA66 using primer pair 1).	Verified presence of insert by PCR using primer pairs 20 and 23. Confirmed sequence of insert by Sanger sequencing using each primer of pair 20 and REV primer of pair 23.	This work
pUA66 P_{alr}-*alr*	Constructed via 2-Fragment Gibson Assembly of P_{alr}-*alr* (PCR-amplified from MG1655 gDNA using primer pair 6) and pUA66 backbone (PCR-amplified from pUA66 using primer pair 1).	Verified presence of insert by PCR using primer pairs 20 and 24. Confirmed sequence of insert by Sanger sequencing using each primer of pair 20.	This work
pUA66 P_{dadAX}-*dadX*	Constructed via 3-Fragment Gibson Assembly of the leading promoter of the *dadAX* operon and *dadX* ORF (PCR-amplified from MG1655 gDNA using primer pairs 7 and 8, respectively) and the pUA66 backbone (PCR-amplified from pUA66 using primer pair 1).	Verified presence of insert by PCR using primer pairs 20 and 25. Confirmed sequence of insert by Sanger sequencing using each primer of pair 20.	This work
pUA66 $P_{dadAX\_dadXRBS}$-*dadX*	Constructed via SDM by PCR-amplification of pUA66 P_{dadAX}-*dadX* using primer pair 9.	Sequence confirmed by Sanger seqeuncing using FWD primer of pair 20.	This work
pGRG25 P_{cycA}-*cycA*	Constructed via 4-Fragment Gibson assmebly of P_{cycA}-*cycA* (amplified from pUA66 P_{cycA}-*cycA* using primer pair 13) and pGRG25 backbone fragments 1, 2, and 3 (amplified from pGRG25 using primer pairs 10, 11, and 12, respectively).	Verified presence of insert by PCR using primer pair 26	This work

pGRG25 P_{ddlA} -*ddlA*	Constructed via 4-Fragment Gibson assmebly of P_{ddlA} -*ddlA* (amplified from pUA66 P_{ddlA} -*ddlA* using primer pair 14) and pGRG25 backbone fragments 1, 2, and 3 (amplified from pGRG25 using primer pairs 10,11, and 12, respectively).	Verified presence of insert by PCR using primer pair 26	This work
pGRG25 P_{ddlB} -*ddlB*	Constructed via 4-Fragment Gibson assmebly of P_{ddlB} -*ddlB* (amplified from pUA66 P_{ddlB} -*ddlB* using primer pair 15) and pGRG25 backbone fragments 1, 2, and 3 (amplified from pGRG25 using primer pairs 10,11, and 12, respectively).	Verified presence of insert by PCR using primer pair 26	This work
pGRG25 P_{alr} -*alr*	Constructed via 4-Fragment Gibson assmebly of P_{alr} -*alr* (amplified from pUA66 P_{alr} -*alr* using primer pair 16) and pGRG25 backbone fragments 1, 2, and 3 (amplified from pGRG25 using primer pairs 10, 11, and 12, respectively).	Verified presence of insert by PCR using primer pair 26	This work
pGRG25 $P_{dadAX\_dadXRBS}$ -*dadX*	Constructed via 4-Fragment Gibson assmebly of $P_{dadAX\_dadX\,\mathrm{RBS}}$-*dadX* (amplified from pUA66 $P_{dadAX\_dadX\,\mathrm{RBS}}$-*dadX* using Primer pair 17) and pGRG25 backbone fragments 1, 2, and 3 (amplified from pGRG25 using primer pairs 10,11, and 12, respectively).	Verified presence of insert by PCR using primer pair 26	This work
pGRG25 P_{N25}-*tetR*	Constructed via 4-Fragment Gibson assmebly of P_{N25}-*tetR* (amplified from gDNA of JR002 using primer pair 18) and pGRG25 backbone fragments 1, 2, and 3 (amplified from pGRG25 using primer pairs 10,11, and 12, respectively).	Verified presence of insert by PCR using primer pair 26	This work
pMAZSK gRNA$_{ddlB}$	Constructed via SDM by PCR-amplification of pMAZSK using primer pair 19.	Sequence verified by Whole Plasmid sequencing by Plasmidsaurus	This work

†Strains listed in Table S3
*Primer pairs listed in Table S4

References

1. Zaslaver A, Bren A, Ronen M, Itzkovitz S, Kikoin I, Shavit S, Liebermeister W, Surette MG, Alon U. 2006. A comprehensive library of fluorescent transcriptional reporters for *Escherichia coli* . *Nat Methods* 3**:** 623–628.

2. McKenzie GJ, Craig NL. 2006. Fast, easy and efficient: site-specific insertion of transgenes into *Enterobacterial* chromosomes using Tn7 without need for selection of the insertion event. *BMC Microbiology* 6: 39.

3. Datsenko KA, Wanner BL. 2000. One-step inactivation of chromosomal genes in *Escherichia coli* K-12 using PCR products. *Proc Natl Acad Sci* U S A 97: 6640–6645.

4. Ronda C, Pedersen LE, Sommer MOA, Nielsen AT. 2016. CRMAGE: CRISPR Optimized MAGE Recombineering. *Scientific Reports* 6:19452.

5. Amato, S.M., Brynildsen, MP. 2014. Nutrient transitions ar a source of persisters in *Echerichia coli* biofilms. *PLoS One* 9: e93110.

6. Robinson, JL, Brynildsen, MP. 2015. An ensemble-guided approach identifies ClpP as a major regulator of transcript levels in nitric oxide-stressed *Escherichia coli* . *Metabolic Engineering* 31: 22-34.

Table S3. List of bacterial strains used in this study.

Strain	Genotype	Description[†]	PCR/Sequence Check(s)[*]	Source
**MG1655**	F^{-} λ^{-} *ilvG*$^{-}$ *rfb*-50 *rph*-*1*	ATCC 700926	N/A	[1]
JR002	MG1655 *attB*::P_{N25}-*tetR*-*gentR*	Used for gDNA extraction for PCR-amplification of P_{N25}-*tetR* for generation of pGRG25-P_{N25}-*tetR*.	N/A	[2]
KS001	MG1655 *attTn7*::MCS	Generated via Tn7 transposition by transformation with pGRG25.	Verified insert integration at attTn7 by colony PCR using primer pair 27.	This work
KS002	MG1655 *attTn7*::P_{cycA}-*cycA*	Generated via Tn7 transposition by transformation with pGRG25 P_{cycA}-*cycA*.	Verified insert integration at attTn7 by colony PCR using primers pair 27 and 28.	This work
KS003	MG1655 *attTn7*::P_{ddlA}-*ddlA*	Generated via Tn7 transposition by transformation with pGRG25 P_{ddlA}-*ddlA*.	Verified insert integration at attTn7 by colony PCR using primers pair 27 and 29.	This work
KS004	MG1655 *attTn7*::P_{ddlB}-*ddlB*	Generated via Tn7 transposition by transformation with pGRG25 P_{ddlB}-*ddlB*.	Verified insert integration at attTn7 by colony PCR using primers pair 27 and 30.	This work
KS005	MG1655 *attTn7*::P_{alr}-*alr*	Generated via Tn7 transposition by transformation with pGRG25 P_{alr}-*alr*.	Verified insert integration at attTn7 by colony PCR using primers pair 27 and 31.	This work
KS006	MG1655 *attTn7*::$P_{dadAX\_dadX\ RBS}$-*dadX*	Generated via Tn7 transposition by transformation with pGRG25 $P_{dadAX\_dadX\ RBS}$-*dadX*.	Verified insert integration at attTn7 by colony PCR using primers pair 27 and 32.	This work
KS007	MG1655 Δ*cycA*	Generated by P1 bacteriophage transduction using Keio collection as donor and MG1655 as recipient strain and curing *kanR* cassette by trasnformation with pCP20.	Verified absence of gene(s) and presence of and presence of scar sequence by colony PCR using primer pairs 34, 35, and 36.	This work
KS008	MG1655 Δ*ddlA*	Generated by P1 Bacteriophage transduction using Keio collection as donor and MG1655 as recipient strain and curing *kanR* cassette by trasnformation with pCP20.	Verified absence of gene(s) and presence of and presence of scar sequence by colony PCR using primer pairs 37, 38, and 39.	This work
KS009	MG1655 Δ*ddlB*	Generated by P1 Bacteriophage transduction using Keio collection as donor and MG1655 as recipient strain and curing *kanR* cassette by trasnformation with pCP20.	Verified absence of gene(s) and presence of and presence of scar sequence by colony PCR using primer pairs 40, 41, and 42.	This work
KS010	MG1655 Δ*alr*	Generated by P1 Bacteriophage transduction using Keio collection as donor and MG1655 as recipient strain and curing *kanR* cassette by trasnformation with pCP20.	Verified absence of gene(s) and presence of and presence of scar sequence by colony PCR using primer pairs 43, 44, 45.	This work
KS011	MG1655 Δ*dadX*	Generated by P1 Bacteriophage transduction using Keio collection as donor and MG1655 as recipient strain and curing *kanR* cassette by trasnformation with pCP20.	Verified absence of gene(s) and presence of and presence of scar sequence by colony PCR using primer pairs 46, 47, 48.	This work
KS012	MG1655 Δ*ddlA* Δ*alr*	Generated by P1 Bacteriophage transduction using Keio collection as donor and MG1655 as recipient strain and curing *kanR* cassette by trasnformation with pCP20.	Verified absence of gene(s) and presence of and presence of scar sequence by colony PCR using primer pairs 37, 38, 39, 43, 44, and 45.	This work
KS013	MG1655 Δ*ddlA* Δ*dadX*	Generated by P1 Bacteriophage transduction using Keio collection as donor and MG1655 as recipient strain and curing *kanR* cassette by trasnformation with pCP20.	Verified absence of gene(s) and presence of and presence of scar sequence by colony PCR using primer pairs 37, 38, 39, 46, 47, and 48.	This work
KS014	MG1655 Δ*ddlB* Δ*alr*	Generated by P1 Bacteriophage transduction using Keio collection as donor and MG1655 as recipient strain and curing *kanR* cassette by trasnformation with pCP20.	Verified absence of gene(s) and presence of and presence of scar sequence by colony PCR using primer pairs 40, 41, 42, 43, 44, and 45.	This work
KS015	MG1655 Δ*ddlB* Δ*dadX*	Generated by P1 Bacteriophage transduction using Keio collection as donor and MG1655 as recipient strain and curing *kanR* cassette by trasnformation with pCP20.	Verified absence of gene(s) and presence of and presence of scar sequence by colony PCR using primer pairs 40, 41, 42, 46, 47, and 48.	This work
**UTI89**	UPEC, cystitis isolate (O18:K1:H7) GenBank:CP000243.1	Urpathogenic *E. coli*	N/A	[3, 4]
KS016	UTI89 *attTn7*::MCS	Generated via Tn7 transposition by transformation with pGRG25.	Verified insert integration at attTn7 by colony PCR using primer pair 27.	This work
KS017	UTI89 *attTn7*::P_{cycA}-*cycA*	Generated via Tn7 transposition by transformation with pGRG25 P_{cycA}-*cycA*.	Verified insert integration at attTn7 by colony PCR using primers pair 27 and 28.	This work

KS018	UTI89 *attTn7* ::P_{N25}-*tetR*	Generated via Tn7 transposition by transformation of UTI89 with pGRG25 P_{N25}-*tetR.*	Verified insert integration at attTn7 by colony PCR using primers pair 27 and 33.	This work
KS019	UTI89 *ddlB(A10T, C22T) attTn7* ::P_{N25}-*tetR*	Generated by CRMAGE of UTI89 *attTn7* ::P_{N25}-*tetR* using pMAZSK $gRNA_{ddlB}$ and mutagentic oligo (5'-gaggaagaacaacatgactgat**T**aaatcgcggtc**T**tgttgggcgggacctccgctgagcgggaagtttct-3')	Sequence confirmed by Sanger seqeuncing using FWD primer of pair 49.	This work
KS020	UTI89 *ddlB(C22T) attTn7* ::P_{N25}-*tetR*	Generated by CRMAGE of UTI89 *attTn7* ::P_{N25}-*tetR* using pMAZSK $gRNA_{ddlB}$ and mutagentic oligo (5'-gaggaagaacaacatgactgataaaatcgcggtc**T**tgttgggcgggacctccgctgagcgggaagtttct-3')	Sequence confirmed by Sanger seqeuncing using FWD primer of pair 49.	This work

† Plasmids listed in Table S2

*Primer pairs listed in Table S4

References

1. Kohanski MA, Dwyer DJ, Hayete B, Lawrence CA, Collins JJ. 2007. A common mechanism of cellular death induced by bactericidal antibiotics. *Cell* 130**:** 797-810.

2. Robinson JL, Brynildsen MP. 2015. An ensemble-guided approach identifies ClpP as a major regulator of transcript levels in nitric oxide-stressed *Escherichia coli. Metabolic Engineering* 31: 22-34.

3. Mulvey MA, Schilling JD, Hultgren SJ. 2001. Establishment of a persistent *Escherichia coli* reservoir during the acute phase of a bladder infection. *Infect Immun* 69**:** 4572-9.

4. Chen SL, Hung CS, Xu J, Reigstad CS, Magrini V, Sabo A, Blasiar D, Bieri T, Meyer RR, Ozersky P, Armstrong JR, Fulton RS, Latreille JP, Spieth J, Hooton TM, Mardis ER, Hultgren SJ, Gordon JI. 2006. Identification of genes subject to positive selection in uropathogenic strains of *Escherichia coli* : a comparative genomics approach. *Proc Natl Acad Sci* U S A 103:5977-82.

Table S4. Oligonucleotides used in this study.

Pair No.	FWD Primer Sequence (5'-3')†	Pair No.	REV Primer Sequence (5'-3')†	Purpose*
1	ctgcaggcatgcaagctc	1	ctcgaggtgaagacgaaaggg	Cloning of pUA66 backbone to generate pUA66 derivatives via Gibson assemblies
2	**CCTTTCGTCTTCACCTCGAG**atgccggtaggttctaaatgg	2	**TAGAGCTTGCATGCCTGCAG**cattgcgccatccagcat	Cloning P_{cycA}-*cycA* from MG1655 gDNA for generation of pUA66 P_{cycA}-*cycA* via Gibson assembly
3	**CCTTTCGTCTTCACCTCGAG**gattaccagcgaaacgtttctc	3	**TAGAGCTTGCATGCCTGCAG**ttacattgtggttttcaatgcgtt	Cloning P_{ddlA}-*ddlA* from MG1655 gDNA for generation of pUA66 P_{ddlA}-*ddlA* via Gibson assembly
4	**CCTTTCGTCTTCACCTCGAG**gccgcgaacaaaaatacgc	4	gccagcctcaccccttat	Cloning P_{ddlB} from MG1655 gDNA for generation of pUA66 P_{ddlB}-*ddlB* via Gibson assembly
5	**GAATAAGGGGTGAGGCTGG**catgactgataaaatcgcggtcc	5	**TAGAGCTTGCATGCCTGCAG**ttagtccgccagttccagaa	Cloning *ddlB* from MG1655 gDNA for generation of pUA66 P_{ddlB}-*ddlB* via Gibson assembly
6	**CCTTTCGTCTTCACCTCGAG**aacaacgtgccgacaaac	6	**TAGAGCTTGCATGCCTGCAG**ttaatccacgtatttcatcgcga	Cloning P_{alr}-alr from MG1655 gDNA for generation of pUA66 P_{alr}-*alr* via Gibson assembly
7	**CCTTTCGTCTTCACCTCGAG**tctttgcgatttcttattatcagagtg	7	agccactccgataacggta	Cloning P_{dadAX} from MG1655 gDNA for generation of pUA66 P_{dadAX}-*dadX* via Gibson assembly
8	**CTACCGTTATCGGAGTGGCT**atgacccgtccgatacagg	8	**TAGAGCTTGCATGCCTGCAG**ttacaccgtcacaaccg	Cloning *dadX* from MG1655 gDNA for generation of pUA66 P_{dadAX}-*dadX* via Gibson assembly
9	**GAAACGAG**atgacccgtccgatacagg	9	**CTTAGCTG**gtagatacagtaaaagaataataatctaatca	Cloning pUA66 P_{dadAX}-*dadX* for generation of pUA66 $P_{dadAX\_dadX\,RBS}$-*dadX* via SDM
10	agattaaatcagaacgcagaagcg	10	gtggcttactaggatccgatatca	Cloning of pGRG25 backbone (fragment 1) to generate pGRG25 derivatives via Gibson assembly
11	tctctcattttgggaggtttgg	11	tctgcgttctgatttaatctgtatca	Cloning of pGRG25 backbone (fragment 2) to generate pGRG25 derivatives via Gibson assembly
12	gccactcgagcacctag	12	aaacctcccaaaatgagagacc	Cloning of pGRG25 backbone (fragment 3) to generate pGRG25 derivatives via Gibson assembly
13	**ATCGGATCCTAGTAAGCCAC**atgccggtaggttctaaatgg	13	**CTCCTAGGTGCTCGAGTGGC**cattgcgccatccagcat	Cloning P_{cycA}-*cycA* from pUA66 P_{cycA}-*cycA* for generation of pGRG25 P_{cycA}-*cycA* via Gibson assembly
14	**ATCGGATCCTAGTAAGCCAC**gattaccagcgaaacgtttctc	14	**CTCCTAGGTGCTCGAGTGGC**ttacattgtggttttcaatgcgtt	Cloning of P_{ddlA}-*ddlA* from pUA66 P_{ddlA}-*ddlA* to generate pGRG25 P_{ddlA}-*ddlA* via Gibson assembly
15	**ATCGGATCCTAGTAAGCCAC**gccgcgaacaaaaatacgc	15	**CTCCTAGGTGCTCGAGTGGC**ttagtccgccagttccagaa	Cloning of P_{ddlB}-*ddlB* from pUA66 P_{ddlB}-*ddlB* to generate pGRG25 P_{ddlB}-*ddlB* via Gibson assembly
16	**ATCGGATCCTAGTAAGCCAC**gaacaacgtgccgacaaac	16	**CTCCTAGGTGCTCGAGTGGC**ttaatccacgtatttcatcgcga	Cloning of P_{alr}-*alr* from pUA66 P_{alrA}-*alr* to generate pGRG25 P_{alr}-*alr* via Gibson assembly
17	**ATCGGATCCTAGTAAGCCAC**tctttgcgatttcttattatcagagtg	17	**CTCCTAGGTGCTCGAGTGGC**ttacaccgtcacaaccgg	Cloning of $P_{dadAX\_dadX\,RBS}$-*dadX* from pUA66 $P_{dadAX\_dadX\,RBS}$-*dadX* to generate pGRG25 $P_{dadAX\_dadX\,RBS}$-*dadX* via Gibson assembly
18	**ATCGGATCCTAGTAAGCCAC**gatagggaatcgattcataaaaaatttatttg	18	**CTCCTAGGTGCTCGAGTGGC**ttaagacccactttcacatttaagttg	Cloning of P_{N25}-*tetR* from JR002 gDNA to generate pGRG25 P_{N25}-*tetR* via Gibson assembly
19	**CCCGCCCAAC**gttttagagctagaaatagcaagttaa	19	**ACCTCCGCTG**gtgctcagtatctctatcact	Cloning of pMAZSK to generate pMAZSK $gRNA_{ddlB}$ via SDM
20	gcgaaacgatcctcatcctg	20	tgcgattctgataacaaactagca	PCR amplification and sequence check of inserts of all pUA66 derivatives
21		21	ccataccggttacaacccag	PCR amplification and sequence check of P_{cycA}-*cycA* insert of pUA66 P_{cycA}-*cycA*
22		22	tctcgttgatcaccacttcg	PCR amplification and sequence check of P_{ddlA}-*ddlA* insert of pUA66 P_{ddlA}-*ddlA*
23		23	cgtccatgctttcagcacta	PCR amplification and sequence check of P_{ddlB}-*ddlB* insert of pUA66 P_{ddlB}-*ddlB*
24		24	accttcgccccataaaat	PCR amplification and sequence check of P_{alr}-*alr* insert of pUA66 P_{alr}-*alr*
25		25	caactcatagcccaccgttc	PCR amplification and sequence check of $P_{dadAX\_dadX\,RBS}$-*dadX* insert of pUA66 $P_{dadAX\_dadX\,RBS}$-*dadX*
26	ggggtggaaatggagttttt	26	cgattaacgagtcgctccat	PCR verification of pGRG25 derivatives
27	gatgacggtttgtcacatgga	27	gatgctggtggcgaagctgt	Colony PCR verification of all insertions at *attTn7*
28		28	ccataccggttacaacccag	Colony PCR verification of P_{cycA}-*cycA* insertion at *attTn7*
29		29	tctcgttgatcaccacttcg	Colony PCR verification of P_{ddlA}-*ddlA* insertion at *attTn7*
30		30	cgtccatgctttcagcacta	Colony PCR verification of P_{ddlB}-*ddlB* insertion at *attTn7*
31		31	accttcgccccataaaat	Colony PCR verification of P_{alr}-*alr* insertion at *attTn7*

32		32	caactcatagcccaccgttc	Colony PCR verification of $P_{dadAX\_dadX\,RBS}$-dadX insertion at *attTn7*
33		33	gacccactttcacatttaag	Colony PCR verification of P_{N25}-*tetR* insertion at *attTn7*
34	tgccggtaggttctaaatgg	34	agctgattctgcaagcgact	Colony PCR check of MG1655 Δ*cycA* (after *kanR* cure)
35		35	atgatggatactttctcggcaggag	Colony PCR check of Keio collection donor (ΔcycA::*kanR*), MG1655 Δ*cycA*::*kanR* (after P1 transduction), and MG1655 Δ*cycA* (after *kanR* cure)
36	ggaattgctgctttcgaatc	36	ccataccggttacaacccag	Colony PCR check of Keio collection donor (Δ*cycA*::*kanR*), MG1655 Δ*cycA*::*kanR* (after P1 transduction), and MG1655 Δ*cycA* (after *kanR* cure)
37	gcaagcttaaataacaactcagca	37	tgtgataccagcggcttatg	Colony PCR check of all MG1655 derivatives involving Δ*ddlA* (after *kanR* cure)
38		38	atgatggatactttctcggcaggag	Colony PCR check of Keio collection donor strain Δ*ddlA*::*kanR* and all MG1655 derivatives involving Δ*ddlA*::*kanR* (after P1 transduction) or Δ*ddlA* (after *kanR* cure)
39	cagacgatcctgcccatatt	39	tctcgttgatcaccacttcg	Colony PCR check of Keio collection donor strain Δ*ddlA*::*kanR* and all MG1655 derivatives involving Δ*ddlA*::*kanR* (after P1 transduction) or Δ*ddlA* (after *kanR* cure)
40	gtaaaagcggtacggcaatg	40	tacagcattggaagcacctg	Colony PCR check of all MG1655 derivatives involving Δ*ddlB* (after *kanR* cure)
41		41	atgatggatactttctcggcaggag	Colony PCR check of Keio collection donor strain Δ*ddlB*::*kanR* and all MG1655 derivatives involving Δ*ddlB*::*kanR* (after P1 transduction) or Δ*ddlB* (after *kanR* cure)
42	ataaaatcgcggtcctgttg	42	cgtccatgctttcagcacta	Colony PCR check of Keio collection donor strain Δ*ddlB*::*kanR* and all MG1655 derivatives involving Δ*ddlB*::*kanR* (after P1 transduction) or Δ*ddlB* (after *kanR* cure)
43	cgataaccgtacgctggaaa	43	aagcccttccatcggtaaat	Colony PCR check of all MG1655 derivatives involving Δ*alr* (after *kanR* cure)
44		44	atgatggatactttctcggcaggag	Colony PCR check of Keio collection donor strain Δ*alr*::*kanR* and all MG1655 derivatives involving Δ*alr*::*kanR* (after P1 transduction) or Δ*alr* (after *kanR* cure)
45	aaagcgaacgcttatggtc	45	accttcgccccataaaat	Colony PCR check of Keio collection donor strain Δ*alr*::*kanR* and all MG1655 derivatives involving Δ*alr*::*kanR* (after P1 transduction) or Δ*alr* (after *kanR* cure)
46	ccggtatccaccattcttga	46	ggttgtcggtgaccaggtag	Colony PCR check of all MG1655 derivatives involving Δ*dadX* (after *kanR* cure)
47		47	atgatggatactttctcggcaggag	Colony PCR check of Keio collection donor strain Δ*dadX*::*kanR* and all MG1655 derivatives involving Δ*dadX*::*kanR* (after P1 transduction) or Δ*dadX* (after *kanR* cure)
48	cacctgcgtacacagcaact	48	caactcatagcccaccgttc	Colony PCR check of Keio collection donor strain Δ*dadX*::*kanR* and all MG1655 derivatives involving Δ*dadX*::*kanR* (after P1 transduction) or Δ*dadX* (after *kanR* cure)
49	atttcgccaatgtgctgacg	49	cgtccatgctttcagcacta	PCR amplification and sequence check of *ddlB* in UTI89 to confirm STOP and/or PAM mutation(s)
50	cgttgacgaatcttggagctcc	50	tcgtcgtttggtatggcttc	Colony PCR amplification to confirm cure of pMA7CR-2.0.
51	gtctggtcctcgagtctggtt	51	gcagtgtgaccgtgtgcttc	Colony PCR amplification to confirm cure of pMAZSK.
52	ctttttcgtgatgcgggcaa	52	ccataccggttacaacccag	qPCR primers for amplification of *cycA* transcripts
53	ggttgcgatactcggtgaaga	53	tcttgtgacgcttccagacc	qPCR primers for amplification of *ddlB* transcripts
54	cggcgaagacttctacagcta	54	caggatggccttggtcaat	qPCR primers for amplification of *phzM* external control RNA [1]

†Text in caps/bold indicate 5' overhang sequences for Gibson assemblies or SDM reactions
* Plasmids listed in Table S2, Strains listed in Table S3

References

1. Robinson, JL, Brynildsen, MP. 2015. An ensemble-guided approach identifies ClpP as a major regulator of transcript levels in nitric oxide-stressed *Escherichia coli*. *Metabolic Engineering* 31: 22-34.

Table S5. Determination of HNIC for each strain used in this study. PTP in bold indicates that the average log(PTP) at that concentration is not significantly different from 0. PTP highlighed in blue indicates PTP at the HNIC.

A) Average PTP on plates containing fosfomycin (FOS)

Strain	FOS concentration (µg/mL)			
	0.1	0.2	0.4	0.8
MG1655	**1.0**	**1.1**	0.3	5E-04
UTI89	N/A	**0.9**	**0.5**	7E-04

B) Average PTP on plates containing tobramycin (TOB)

Strain	TOB concentration (µg/mL)			
	0.128	0.256	0.512	1.024
MG1655	**1.0**	**0.7**	9E-03	2E-06
UTI89	**0.9**	**0.7**	7E-02	1E-04

C) Average PTP on plates containing ciprofloxacin (CIP)

Strain	CIP concentration (µg/mL)			
	0.001	0.002	0.004	0.008
MG1655	**1.0**	**0.6**	0.3	2E-02
UTI89	N/A	**0.9**	**0.8**	0.4

D) Average PTP on plates containing D-cycloserine (DCS)

MG1655 and derivatives*

Strain	DCS concentration (µg/mL)			
	0.064	0.128	0.256	0.512
MG1655	**0.9**	**0.7**	0.3	2E-02
MG1655 Δ*ddlB*	**0.8**	**0.4**	2E-02	5E-04
MG1655 Δ*ddlA*	**0.9**	**0.9**	**0.4**	7E-02
MG1655 Δ*dadX*	**1.0**	**0.9**	**0.3**	2E-02
MG1655 Δ*alr*	**0.9**	**0.7**	**0.3**	4E-02
MG1655 Δ*ddlB* Δ*dadX*	**0.6**	0.1	5E-04	2E-04
MG1655 Δ*ddlB* Δ*alr*	**0.7**	**0.2**	9E-03	4E-04
MG1655 Δ*ddlA* Δ*dadX*	**1.0**	**0.6**	0.1	1E-03
MG1655 Δ*ddlA* Δ*alr*	**0.9**	**0.7**	**0.2**	6E-03
MG1655 attTn7::MCS	**1.0**	**0.8**	**0.5**	0.2
MG1655 *attTn7*::P_{cycA}-*cycA*	**0.9**	**0.9**	**0.4**	9E-02
MG1655 *attTn7*::P_{ddlB}-*ddlB*	**1.0**	**0.9**	**0.9**	**0.5**
MG1655 *attTn7*::P_{ddlA}-*ddlA*	**0.9**	**0.9**	**0.7**	**0.4**
MG1655 *attTn7*::$P_{dadAX\_dadX\ RBS}$-*dadX*	**0.9**	**1.0**	**0.7**	**0.2**
MG1655 *attTn7*::P_{alr}-*alr*	**1.0**	**0.8**	**0.5**	0.1

*Δ*cycA* excluded from table, as average log(PTP) was not significantly different from 0 at all DCS concentrations investigated in this study (HNIC ≥ 2.048 µg/mL)

UTI89 and derivatives

Strain	DCS concentration (µg/mL)			
	0.256	0.512	1.024	2.048
UTI89	**0.9**	**0.9**	0.2	2E-02
UTI89 *attTn7* :::MCS	**0.8**	**0.9**	**0.3**	1E-02
UTI89 *attTn7* ::P_{cycA} -*cycA*	**1.0**	**0.7**	0.2	3E-03
UTI89 *ddlB* (C22T) *attTn7* ::P_{N25}-*tetR*	**1.0**	**0.9**	0.4	7E-04
UTI89 *ddlB* (A10T,C22T) *attTn7* ::P_{N25}-*tetR*	**0.6**	0.2	9E-04	2E-05
